# Mortality prediction in patients with acute myocardial infarction and prior ischemic stroke: a prospective cohort study with explainable machine learning and SHAP analysis

**DOI:** 10.3389/fcvm.2026.1949226

**Published:** 2026-10-27

**Authors:** Congcong Han, Abudureheman Muladili, Ning Song, Chunfang Shan, Fen Liu, Xiaomei Li, Yining Yang, Qian Zhao

**Affiliations:** 1 Department of Cardiology, First Affiliated Hospital of Xinjiang Medical University, Urumqi, China; 2 School of Public Health, Xinjiang Medical University, Urumqi, China; 3 Department of Cardiology, People's Hospital of Xinjiang Uyghur Autonomous Region, Urumqi, China

**Keywords:** acute myocardial infarction, machine learning, mortality, percutaneous coronary intervention, stroke

## Abstract

**Background:**

Patients with acute myocardial infarction (AMI) and a prior history of ischemic stroke (IS) represent a high-risk population with limited prognostic tools. This study aimed to develop and validate an interpretable machine learning model for predicting all-cause mortality in AMI patients with prior IS.

**Methods:**

This prospective single-center cohort included 543 consecutive patients with AMI and prior IS who underwent Percutaneous Coronary Intervention (PCI). Candidate predictors were screened using univariable Cox regression, Boruta, and LASSO Cox regression. Six survival models were compared: Cox, elastic-net Cox, random survival forest (RSF), GBM survival, XGBoost survival, and Survival SVM. Internal validation used 100 bootstrap resamples with out-of-bag (OOB) evaluation. Performance was evaluated using Harrell's C-index, IPCW time-dependent AUC, calibration measures, Brier score, and decision-curve analysis. SHAP-based and permutation analyses were used for model interpretation.

**Results:**

During a median follow-up of 3.03 years, 66 patients (12.2%) died. Six predictors were selected: age, hemoglobin, prognostic nutritional index, estimated glomerular filtration rate, atherogenic index of plasma, and monocyte-to-lymphocyte ratio. Although RSF achieved the numerically highest OOB C-index, elastic-net Cox showed comparable discrimination, better calibration, a lower 3-year Brier score, and greater interpretability and was selected as the final model. Its OOB C-index was 0.714 (95% CI 0.616–0.790), and its 1-, 2-, and 3-year IPCW AUCs were 0.745, 0.704, and 0.701, respectively. Age and hemoglobin made the largest contributions to model predictions. Elastic-net Cox showed numerically better performance than GRACE, but statistical superiority in discrimination was not demonstrated.

**Conclusions:**

The six-variable elastic-net Cox model provided moderate discrimination and relatively favorable calibration. Independent external validation and prospective implementation studies are required before clinical use.

## Introduction

Cardiovascular disease remains the leading cause of global mortality and disability, with acute myocardial infarction (AMI) representing one of the conditions with the highest disease burden (1). A substantial proportion of patients presenting with AMI carry a prior history of ischemic stroke (IS), reflecting the shared atherosclerotic and thrombotic substrates linking the cerebral and coronary circulations. When AMI and IS occur simultaneously or in close succession—a syndrome termed cardiocerebral infarction—the condition is rare but devastating, affecting approximately 1.3% of patients in population-based registries and carrying a 2- to 3-fold increased risk of 30-day mortality (2). However, AMI occurs more frequently in patients with a remote history of IS or persistent post-stroke sequelae, and among patients with AMI, those with prior IS have a significantly worse prognosis than those without a history of stroke (3).

The poor prognosis of patients with myocardial infarction complicated by cerebral infarction stems from the convergence of chronic cerebrovascular injury and acute myocardial ischemia (4–7). Shared atherosclerotic substrates, systemic inflammatory cascades (5), renal impairment (6), and metabolic disturbances create a multi-morbid state in which these pathologies interact in a non-linear fashion. Consequently, conventional risk stratification tools, such as the Global Registry of Acute Coronary Events (GRACE) score and the TIMI risk score, evaluate single-organ systems in isolation and fail to capture these multi-system interactions (8). Accurate, individualized prognostic assessment is therefore urgently needed to guide risk stratification and optimize clinical decisions in this volatile population. In recent years, machine learning (ML) has emerged as a powerful paradigm in medical prognostics (9), frequently outperforming traditional statistical methods (10). This superiority is largely driven by ML's non-parametric nature and its capacity to model highly complex, non-linear associations among variables derived from intricate clinical data (11–14). However, existing ML frameworks have predominantly focused on predicting outcomes for AMI or stroke independently (15, 16). Evidence leveraging ML to predict mortality specifically for patients with AMI and a prior history of IS—particularly those undergoing percutaneous coronary intervention (PCI)—remains scarce. To address this knowledge gap, this study utilizes a prospective cohort to develop and validate an interpretable machine learning model for predicting death in patients with patients with AMI and a prior history of IS, thereby facilitating early, individualized risk stratification at the bedside.

## Methods

### Participants

We conducted a prospective cohort study that consecutively enrolled patients admitted to the First Affiliated Hospital of Xinjiang Medical University for chest pain between February 2015 and June 2023, who were diagnosed with AMI with a prior history of IS and underwent PCI within 12 h of chest pain onset. All participants provided written informed consent. The study protocol was approved by the Ethics Committee of the First Affiliated Hospital of Xinjiang Medical University (20140116-03) and complied with the Declaration of Helsinki. This study has been registered in the Chinese Clinical Trial Registry (ChiCTR2400087256). Definitions of AMI subtypes are provided in Supplementary Methods.

During the study period, a total of 2,368 patients presenting with chest pain were screened. Of these, 1,794 did not meet the diagnostic criteria for AMI with a prior history of IS, leaving 574 eligible patients. Among them, 18 patients were excluded according to the exclusion criteria: severe valvular or pericardial diseases (n = 6), severe infection, malignant tumor, or severe organ failure (*n* = 5), expected lifespan < 1 year (*n* = 1), critically missing clinical data (*n* = 5), and age < 18 years (*n* = 1). During follow-up, 13 patients (2.4%) were lost to follow-up and censored at the date of their last successful contact. Finally, 543 patients constituted the final study population. The patient-selection flow is presented in Figure 1.

**Figure 1:**
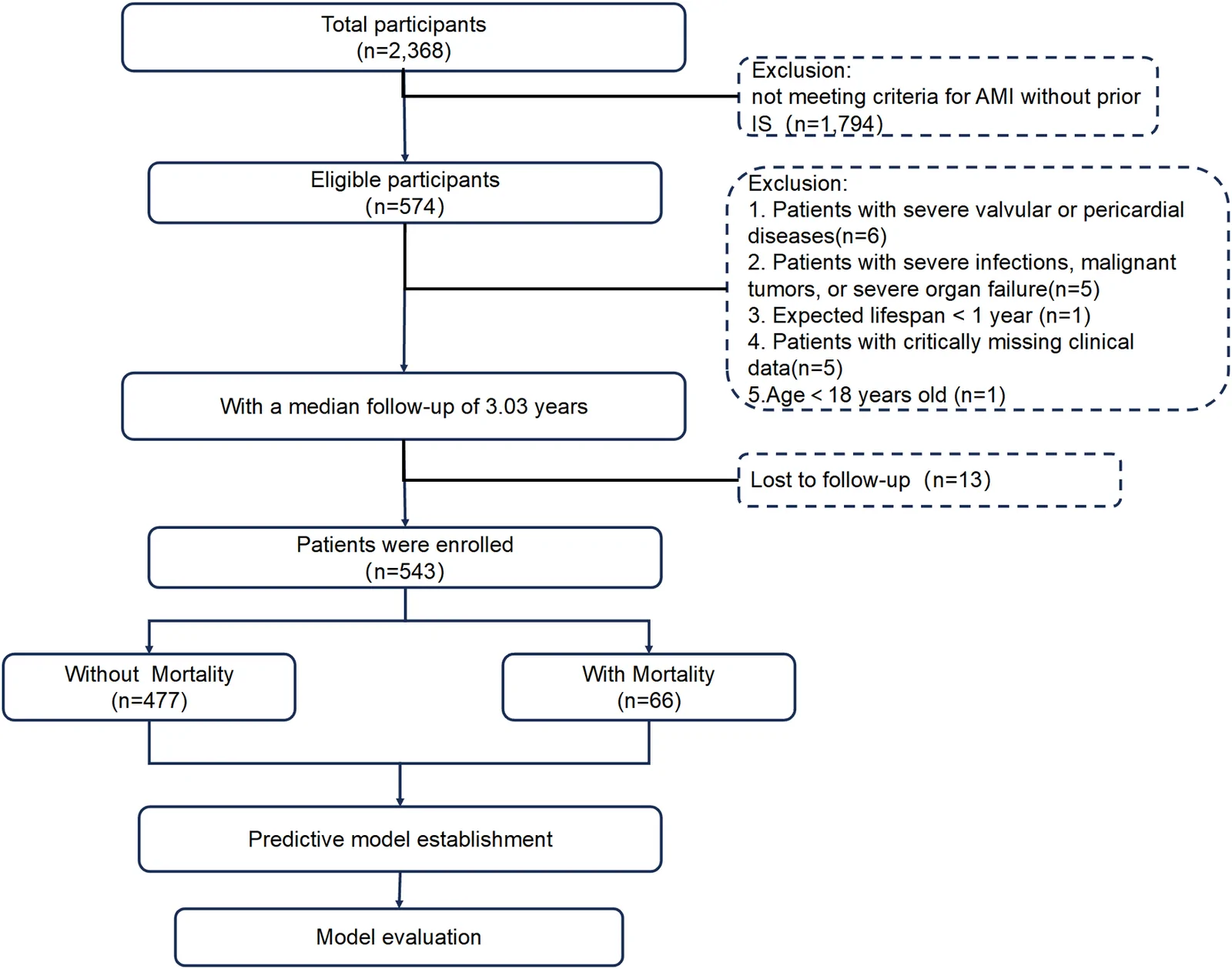
Flowchart of the research.

The primary inclusion criteria were as follows: (1) Meeting the diagnostic criteria for AMI and IS; (2) patients without severe infection, malignant tumor, or severe organ failure; (3) Age ≥ 18 years old. The key exclusion criteria included the following: (1) Patients with severe valvular or pericardial diseases; (2) Expected lifespan < 1 year; (3) Patients with critically missing clinical data.

### Data collection

Baseline clinical data were collected as follows: (1) demographic characteristics and medical history (age, sex, smoking, alcohol consumption, history of hypertension and diabetes) and (2) physical examination findings (height, weight, and blood pressure) were obtained at admission. (3) Laboratory tests were performed using blood samples collected within 12 h of symptom onset and before revascularization. Routine hematologic examinations included red blood cell count, hematocrit, hemoglobin, and white blood cell count. Lipid profiles included total cholesterol, triglycerides (TG), high-density lipoprotein cholesterol (HDL-C), and low-density lipoprotein cholesterol (LDL-C). Glycemic status was assessed by fasting plasma glucose and glycated hemoglobin (HbA1c). Renal function was evaluated by urea, creatinine, and estimated glomerular filtration rate (eGFR); liver function was assessed by alanine aminotransferase (ALT), total bilirubin, direct bilirubin, total protein, and albumin. Inflammatory markers included interleukin-6 (IL-6) and procalcitonin. N-terminal pro-B-type natriuretic peptide (NT-proBNP) was also measured. Except for echocardiography, all baseline variables were therefore collected before PCI and initiation of medical treatment.(4) Cardiac function was assessed by echocardiography within 72 h of admission and before discharge, generally after hemodynamic stabilization; these assessments reflect the early post-admission stable state and are not affected by the PCI procedure itself.

### Definition and calculation of relevant indicators

Patients who smoked ≥1 cigarette per day before hospitalization were defined as smokers (17). Hypertension was defined as meeting one or more of the following criteria: (1) Systolic blood pressure (SBP) ≥ 140 mmHg; (2) Diastolic blood pressure (DBP) ≥ 90 mmHg; (3) a self-reported history of physician-diagnosed hypertension; or (4) current use of antihypertensive medication (18). Diabetes was diagnosed according to established criteria, meeting at least one of the following: a fasting plasma glucose level ≥7.0 mmol/L, a 2-hour plasma glucose level ≥11.1 mmol/L during a 75-g oral glucose tolerance test, a HbA1c level ≥6.5%, or a random plasma glucose level ≥11.1 mmol/L in the presence of classic hyperglycemic symptoms (19). The diagnosis of ischemic stroke was made according to the 2026 Guidelines for the Early Management of Acute Ischemic Stroke (American Heart Association/American Stroke Association) (20). Given the shared atherosclerotic, metabolic, and inflammatory pathways underlying AMI and IS, we calculated and integrated several validated composite indices reflecting dyslipidemia, inflammation, nutritional status, and renal function. These indices included the atherogenic index of plasma (AIP), prognostic nutritional index (PNI), monocyte-to-lymphocyte ratio (MLR), and eGFR, calculated as follows:

BMI, Weight (kg)/Height^2^ (m^2^); AIP, log_10_(TG/HDL-C); MLR, Monocyte count/Lymphocyte count; PNI, Serum albumin (g/L) + 5 × Lymphocyte count (  × 10⁹/L); eGFR, 175 × (Serum creatinine [mg/dL])^−1.154^ × Age^−0.203^ × (0.742 if female), where serum creatinine (mg/dL) = serum creatinine (µmol/L) × 0.01131. eGFR was expressed in mL/min/1.73 m^2^.

### Follow-up and endpoints

The primary outcome was death during follow-up. The time origin was defined as the date of hospital discharge, and survival time was calculated from discharge, with death or censoring as the endpoint. All patients were followed up by telephone or outpatient visit every 12 months after discharge, and relevant information was collected and verified through medical record review and telephone contact with the patients and/or their family members. Loss to follow-up was defined as being unable to contact the patient at any scheduled follow-up or voluntary withdrawal from the study, and the censoring time for these patients was the date of their last successful contact. The end of the follow-up period for this study was fixed at December 31, 2023. A total of 543 patients were included in this cohort, with a median follow-up of 3.03 years; during follow-up, 66 deaths occurred, corresponding to a mortality rate of 12.2%.

### Missing data handling

Missing data were handled using multiple imputation by chained equations (MICE) with the mice R package (version 3.19.0). Variables with more than 15% missing values were excluded before imputation. The proportion of missing values for each remaining candidate predictor is reported in Supplementary Table S1 (range, 0%–14.0%). Continuous variables with missing values were imputed using predictive mean matching (PMM), whereas binary variables were imputed using logistic regression (logreg). Follow-up time and vital status were not imputed but were included as auxiliary predictors in the imputation model used for the full-data analysis. Twenty imputed datasets were generated (m = 20), with 20 iterations per imputation chain. Convergence was assessed by examining trace plots of the chain means and standard deviations, and no systematic trends or persistent separation between chains were observed (Supplementary Table S1).

To reduce information leakage during internal validation, imputation was re-estimated separately within each Bootstrap training sample. The ignore argument was used to ensure that the corresponding out-of-bag (OOB) observations did not contribute to estimation of the imputation model, although missing predictor values in those observations were subsequently imputed using relationships estimated from the Bootstrap training sample. In the Bootstrap validation procedure, the predictor matrix was constrained so that follow-up time and vital status could not be used to impute predictor values. Composite indices, including PNI, AIP, and MLR, were recalculated within each completed dataset after imputation to preserve imputation uncertainty.

### Feature selection

Univariable Cox regression was used for initial screening of candidate predictors, with a threshold of *P* < 0.10 to avoid prematurely discarding potentially weakly associate.

ed variables. least absolute shrinkage and selection operator (LASSO) Cox regression was used for variable selection and to reduce the influence of multicollinearity through L_1_ regularization. The penalty parameter *λ* was determined by 5-fold cross-validation using the lambda.1se criterion, defined as the largest value of *λ* for which the cross-validated error was within one standard error of the minimum. This criterion generally favors a more parsimonious predictor set than lambda.min (21). The Boruta algorithm is a random forest-based wrapper-type feature selection method that identifies all features carrying meaningful predictive information by comparing them against permuted shadow features, and is well suited to capturing non-linear relationships and complex interactions that conventional Cox regression may overlook (22, 23).

We employed a hybrid feature-selection strategy that integrated the complementary strengths of univariable Cox regression, LASSO Cox regression, and the Boruta algorithm (24–26). To reduce the risk of overfitting and false-positive selection, only variables consistently identified by all three methods were retained in the final model. The number of final predictors was restricted so that the events per variable (EPV) was at least 10. Multicollinearity among the final predictors was assessed by calculating variance inflation factor (VIF) values and the correlation matrix; VIF values > 5 were considered indicative of potential multicollinearity.

### Model development

We developed and compared six survival prediction models: a Cox proportional hazards model, an elastic-net penalized Cox model, a random survival forest (RSF), a gradient boosting machine (GBM) survival model, an extreme gradient boosting (XGBoost) survival model, and a survival support vector machine (Survival SVM). The six predictors were fixed before internal validation, and feature selection was not repeated within the bootstrap resamples.

Except for the standard Cox model, model-specific hyperparameters were optimized within each bootstrap training sample. Five-fold cross-validation was used to select the elastic-net mixing parameter *α*, RSF mtry and nodesize, XGBoost maximum tree depth, learning rate and number of boosting rounds, and Survival SVM gamma.mu and radial basis function (RBF) kerne parameter sigma^2^, using the mean Harrell C-index across validation folds. For the elastic-net Cox model, *λ* was determined by an additional internal cross-validation procedure using the lambda.1se criterion. For GBM, interaction depth and shrinkage were compared using five-fold cross-validation, whereas the effective number of boosting iterations was determined from the out-of-bag error within the training data. The number of trees in the RSF was fixed at 300.

Internal validation was performed using 100 Bootstrap-OOB resamples. In each resample, a bootstrap training sample was drawn with replacement from the original development cohort, and patients not selected constituted the corresponding OOB evaluation sample. Missing-data imputation was re-estimated within each bootstrap resample. The training and OOB observations were temporarily combined to generate completed OOB predictor values, but the ignore argument in the mice package ensured that OOB observations did not contribute to estimation of the imputation models. Follow-up time and vital status were excluded from the predictor matrix used to impute predictor values, and were not themselves imputed. One completed dataset was generated in each bootstrap resample, and PNI, AIP, and MLR were recalculated after imputation.

Hyperparameter tuning, estimation of standardization parameters, and model fitting were subsequently performed using only the bootstrap training sample. The imputation relationships and standardization parameters estimated from the training sample were then applied to the corresponding OOB sample. OOB outcomes and follow-up times were used solely for performance evaluation and did not contribute to imputation-model estimation, standardization, hyperparameter selection, or model fitting, thereby minimizing information leakage. After internal validation, the final model was refitted separately in each of the 20 multiply imputed full-development datasets, and predictions were averaged across the 20 fitted models to obtain ensemble predictions.

### Model evaluation

Model performance was evaluated in terms of discrimination, calibration, overall prediction error, and clinical utility. Harrell's concordance index (C-index) was used as the primary discrimination metric, with higher values indicating better discrimination. Inverse probability of censoring weighting (IPCW) time-dependent AUCs at 1, 2, and 3 years were additionally calculated to evaluate discrimination at different prediction horizons. Given the relatively low event rate, IPCW time-dependent precision-recall curves and their corresponding areas under the precision-recall curve (PR-AUCs) were used as supplementary performance measures.

Calibration was assessed using 1-, 2-, and 3-year calibration curves based on patient-level averaged OOB predictions. Patients were divided into five approximately equal-sized groups according to their predicted risks. Within each group, the mean predicted mortality risk was compared with the observed mortality risk estimated using the Kaplan–Meier method, with Greenwood 95% confidence intervals. The 3-year calibration intercept and calibration slope were also reported. Overall prediction error at 3 years was assessed using the IPCW Brier score, with lower values indicating better overall predictive performance. IPCW decision curve analysis was used to evaluate clinical net benefit at 3 years across risk thresholds ranging from 1% to 30%, with the treat-all and treat-none strategies as references.

The final model was selected primarily according to the bootstrap OOB C-index of the fixed six-predictor models. Models with a mean C-index within an absolute difference of 0.02 from the highest value were considered to have broadly comparable discrimination. Among these models, selection prioritized, in order, a calibration slope closest to 1, a lower 3-year IPCW Brier score, and, if necessary, a higher C-index. Pairwise comparisons of IPCW time-dependent AUCs at 1, 2, and 3 years were performed using patient-level averaged OOB predictions. AUC differences, 95% confidence intervals, and Holm-adjusted *P* values were reported. Because the final model was an elastic-net Cox model, an unpenalized Cox reference model containing the same six predictors was fitted, and the proportional hazards assumption was assessed using scaled Schoenfeld residuals.

The final model was compared with the GRACE risk score using the C-index and 3-year IPCW time-dependent AUC in the same bootstrap OOB samples, with paired differences and 95% confidence intervals reported. Three-year calibration curves and IPCW decision curves were further compared between the final model and the recalibrated GRACE model in the development cohort. Continuous net reclassification improvement (NRI) and integrated discrimination improvement (IDI) at 3 years, with 95% confidence intervals, were used to explore the incremental predictive value of adding the final-model risk score to the GRACE score. Because the GRACE calibration and decision-curve comparisons, as well as the NRI/IDI analyses, were conducted using the full development cohort, these analyses were regarded as supplementary and exploratory.

### Model interpretability

The final elastic-net Cox model was interpreted using the survex (version 1.2.0), kernelshap (version 0.9.1), and shapviz (version 0.10.4) packages in R. Kernel SHAP (SHapley Additive exPlanations) was applied to the mean standardized risk score obtained from the ensemble of models fitted across 20 multiply imputed datasets, and global feature importance was quantified using the mean absolute Shapley additive explanations (SHAP) value (27). SurvSHAP(t) was further used to explain the time-varying contributions of predictors to the predicted risk of death over 0–3 years; positive values indicated an increase in the predicted risk of death, whereas negative values indicated a decrease. Time-dependent permutation importance and survival partial dependence plots were used to evaluate the contributions of individual predictors to model performance and to predicted survival probabilities, respectively. All interpretation analyses were performed on the complete development cohort after the final model was refitted. These analyses were not part of the bootstrap OOB validation and did not contribute to model training, selection, or performance evaluation. Given the potential correlations among predictors, SHAP results were interpreted as model-based predictive attributions rather than causal effects or independent associations.

### Statistical analysis

Statistical analyses were performed using Stata (version 17.0) and R software (version 4.6.1). Categorical variables were presented as frequencies and percentages, and analyzed with Chi-square test. Normality of continuous variables was assessed using Q-Q plots. Normally distributed variables are expressed as mean and standard deviation (SD) and analyzed with t-tests; non-normally distributed variables are presented as median (interquartile range, IQR) and analyzed using Wilcoxon rank-sum tests for group comparisons. A two-tailed *P*-value < 0.05 was considered statistically significant.

## Result

### Characteristics of the studied cohort

A total of 574 patients with AMI complicated by IS were initially enrolled in this study. Of these, 18 patients were excluded for not meeting the inclusion and exclusion criteria, and 13 patients were lost to follow-up during the follow-up period. Ultimately, 543 patients were included in the study cohort (Figure 1). The mean age of patients in this cohort was (61.0 ± 12.6) years, and 447 (82.3%) were male.

The median follow-up duration was 3.03 years (IQR 1.63–4.24), with a mortality rate of 12.2% (66/543). Compared with surviving patients, non-survivors were older (mean: 69.8 ± 13.2 vs. 59.8 ± 12.0 years, *P* < 0.001) and had a lower smoking rate (27.3% vs. 50.5%, *P* < 0.001); no statistically significant differences were observed between the two groups in terms of sex, BMI, history of alcohol consumption, hypertension, and diabetes (all *P* > 0.05). Non-survivors had higher levels of inflammation and poorer nutritional status, with significantly decreased red blood cell count, hemoglobin, hematocrit, lymphocyte percentage and count, and PNI (all *P* < 0.05), while red blood cell distribution width, mean corpuscular volume, neutrophil percentage, eosinophil count, as well as MLR were significantly increased (all *P* < 0.05) (Table 1).

**Table 1:** Baseline characteristics of the study group.

Characteristics	All patients (*n* = 543)	Without mortality (*n* = 477)	With mortality (*n* = 66)	*P*-value
Age (years)	61.0 ± 12.6	59.8 ± 12.0	69.8 ± 13.2	<0.001
Male (*n*, %)	447 (82.3)	396 (83.0)	51 (77.3)	0.251
Smoking (*n*, %)	259 (47.7)	241 (50.5)	18 (27.3)	<0.001
Drinking (*n*, %)	82 (15.1)	76 (15.9)	6 (9.1)	0.092
Hypertension (*n*, %)	288 (53.0)	248 (52.0)	40 (60.6)	0.189
Diabetes (*n*, %)	157 (28.9)	133 (27.9)	24 (36.4)	0.154
SBP (mmHg)	124.9 ± 21.3	124.8 ± 20.7	125.8 ± 25.0	0.722
DBP (mmHg)	77.1 ± 14.0	77.5 ± 13.8	74.4 ± 15.2	0.093
BMI (kg/m^2^)	25.9 ± 3.6	25.9 ± 3.5	25.4 ± 4.5	0.303
Heartrate (bpm)	83.3 ± 15.5	83.3 ± 15.3	83.8 ± 17.3	0.812
WBC ( × 10^9^/L)	10.4 ± 3.6	10.3 ± 3.6	10.5 ± 3.7	0.743
RBC ( × 10^9^/L)	4.8 ± 0.6	4.8 ± 0.6	4.5 ± 0.7	<0.001
MCV (fL)	90.2 ± 5.1	90.1 ± 4.8	91.4 ± 6.8	0.043
RDW (%)	12.9 ± 0.91	12.8 ± 0.88	13.2 ± 1.02	<0.001
Platelet count ( × 10^9^/L)	230.5 ± 66.4	230.7 ± 63.8	228.6 ± 83.2	0.810
Lymphocyte percentage (%)	18.9 ± 10.9	19.4 ± 11.2	15.6 ± 8.5	0.009
Lymphocyte count ( × 10^9^/L)	1.8 ± 1.2	1.9 ± 1.2	1.6 ± 1.1	0.048
Monocyte percentage (%)	6.0 ± 2.4	6.0 ± 2.3	6.2 ± 3.1	0.351
Monocyte count ( × 10^9^/L)	0.6 ± 0.3	0.6 ± 0.3	0.6 ± 0.4	0.372
Neutrophil percentage (%)	73.8 ± 12.6	73.3 ± 12.8	77.1 ± 10.4	0.023
Neutrophil ( × 10^9^/L)	7.8 ± 3.5	7.8 ± 3.5	8.2 ± 3.3	0.370
Hematocrit (%)	41.6 ± 9.7	42.1 ± 9.3	37.5 ± 11.9	<0.001
Eosinophil percentage (%)	0.5 (0.2,1.3)	0.6 (0.2,1.4)	0.3 (0.2,0.9)	0.038
Basophil percentage (%)	0.3 (0.2,0.5)	0.3 (0.2,0.5)	0.3 (0.2,0.5)	0.410
Eosinophil count ( × 10^9^/L)	0.1 (0.0,0.1)	0.1 (0.0,0.1)	0.0 (0.0,0.1)	0.042
Basophil count ( × 10^9^/L)	0.0 (0.0,0.0)	0.0 (0.0,0.0)	0.0 (0.0,0.0)	0.348
Hemoglobin (g/L)	145.9 ± 18.9	147.4 ± 18.5	135.5 ± 19.1	<0.001
TC (mmol/L)	4.6 ± 1.2	4.6 ± 1.2	4.4 ± 1.5	0.135
TG (mmol/L)	1.7 (1.2,2.5)	1.7 (1.2,2.6)	1.3 (0.8,2.3)	0.002
HDL-C (mmol/L)	0.9 ± 0.2	0.9 ± 0.2	1.0 ± 0.3	0.330
LDL-C (mmol/L)	2.9 ± 1.0	2.9 ± 1.0	2.7 ± 1.2	0.162
Urea (*μ*mol/L)	6.3 ± 2.6	6.2 ± 2.4	7.3 ± 3.6	0.001
Creatinine (μmol/L)	83.8 ± 60.0	81.8 ± 58.6	98.0 ± 68.2	0.004
Total Bilirubin (μmol/L)	15.0 ± 6.7	15.0 ± 6.8	15.1 ± 6.2	0.908
Direct Bilirubin (μmol/L)	0.3 (0.3,1.8)	0.3 (0.3,1.6)	1.3 (0.3,3.1)	<0.001
Total Protein (g/L)	72.9 ± 6.9	73.1 ± 6.8	71.2 ± 7.5	0.035
Albumin (g/L)	41.4 ± 4.2	41.7 ± 4.1	39.0 ± 4.7	<0.001
AST (U/L)	38.6 (25.8,82.6)	38.6 (26.0,82.6)	36.6 (24.6,76)	0.387
ALT (U/L)	28.2 (21.0,43.3)	29.0 (21.7,43.8)	23.0 (19.5,34.0)	0.025
IL-6 (pg/mL)	7.5 (4.6,16.2)	7.4 (4.5,15.1)	11.4 (6.0,42.0)	0.004
Procalcitonin (ng/mL)	0.0 (0.0,0.1)	0.0 (0.0,0.1)	0.1 (0.0,0.1)	0.001
Blood Glucose (mmol/L)	8.0 ± 4.0	8.0 ± 4.0	8.3 ± 3.7	0.560
D-Dimer (ng/mL)	128.0 (82.0,213.0)	121.0 (80.0,196.0)	225.5 (135.0,398.0)	<0.001
HbA1c (%)	6.7 ± 1.7	6.6 ± 1.6	7.3 ± 1.8	0.001
eGFR (mL/min/1.73m^2^)	96.4 ± 33.5	98.2 ± 32.7	83.7 ± 36.4	<0.001
LVEF (%)	56.2 ± 8.8	56.4 ± 8.8	54.9 ± 8.8	0.187
PNI	50.6 ± 7.9	51.1 ± 7.9	46.8 ± 7.1	<0.001
AIP	0.3 (0.0,0.5)	0.3 (0.1,0.5)	0.2 (0.0,0.4)	0.013
MLR	0.4 ± 0.3	0.4 ± 0.3	0.5 ± 0.3	0.024

SBP, systolic blood pressure; DBP, diastolic blood pressure; BMI, body mass index; WBC, white blood cell count; RBC, red blood cell; MCV, mean corpuscular volume; RDW, red blood cell distribution width; TC, total cholesterol; TG, triglyceride; HDL-C, high-density lipoprotein cholesterol; LDL-C, low-density lipoprotein cholesterol; AST, Aspartate Aminotransferase; ALT, alanine aminotransferase; IL-6, interleukin-6; NT-ProBNP, n-terminal pro-B-type natriuretic peptide; HbA1c, haemoglobin A1c; eGFR, estimated glomerular filtration rate; LVEF, left ventricular ejection fraction

Non-survivors showed significant differences in metabolic, hepatic, renal, and cardiac biomarkers. They had lower TG, albumin, total protein, alanine aminotransferase, eGFR, and AIP levels, but higher HbA1c, NT-proBNP, D-dimer, procalcitonin, and IL-6 levels than survivors.

### Feature selection

The proportions of missing data among the candidate variables ranged from 0% to 14.0%. A total of 20 imputed datasets were generated. Trace plots of the imputation chains showed no systematic trends or persistent separation between chains, indicating stable convergence (Supplementary Figure S1).

Univariable Cox regression identified 13 variables that met the prespecified threshold (*P* < 0.10), including age, hemoglobin, HbA1c, hematocrit, PNI, BMI, eGFR, AIP, MLR, DBP, neutrophil percentage, procalcitonin, and TC (Supplementary Table S2).

The Boruta algorithm iteratively eliminated statistically insignificant features and identified features with strong and weak relevance to the outcome. After 100 iterations, 10 variables were confirmed as important: age, hemoglobin, PNI, hematocrit, MLR, White Blood Cell, procalcitonin, ALT, eGFR, and AIP. Supplementary Table S3 summarizes the importance statistics for each variable in the Boruta analysis, including mean, median, minimum, and maximum importance scores, as well as the final decision.

In the LASSO model with five-fold cross-validation, 13 clinical features had nonzero coefficients at the optimal *λ* value (*λ*_min). At this optimal penalty level, LASSO regression identified 13 significant variables: age, hemoglobin, BMI, WBC, PNI, AIP, MLR, eGFR, DBP, LDL-C, total bilirubin, sex, and HbA1c (Supplementary Table S4). Supplementary Table S4 presents the regression coefficients of each variable at both the optimal *λ* (λ_min) and the most parsimonious *λ* (λ_1se), as well as the selection status of each variable.

Variables consistently identified by all three methods were retained as the final predictors (Figure 2). As shown in the Venn diagram (Supplementary Figure S2) and Supplementary Table S5, six variables—age, hemoglobin, PNI, MLR, eGFR, and AIP—were selected by all three methods and included in the final prediction model. No significant multicollinearity was observed among the six predictors (Supplementary Figure S3, Supplementary Table S6).

**Figure 2:**
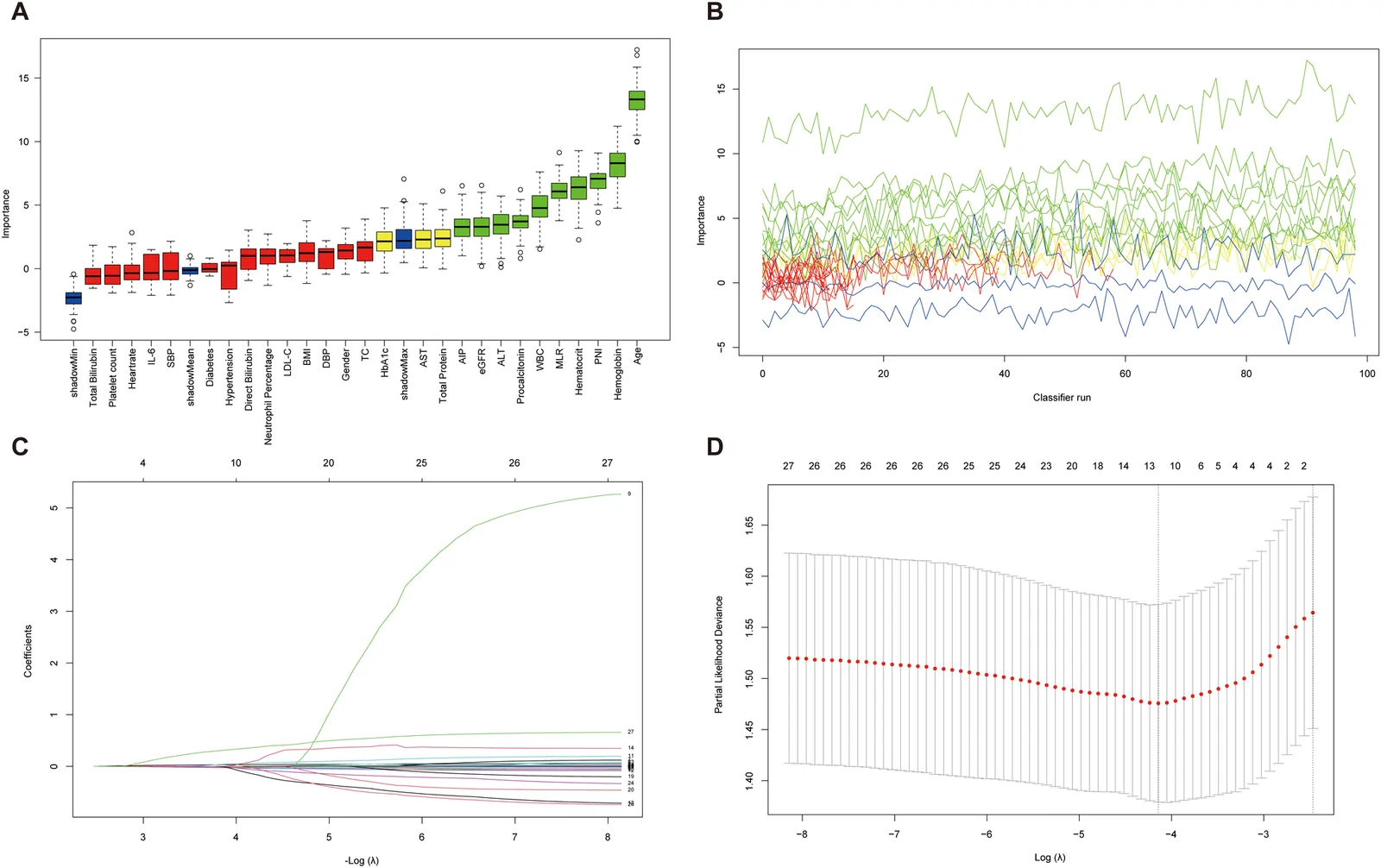
Feature selection using boruta and LASSO cox regression. **(A)** Boruta importance plot: green, yellow, and red indicate confirmed, tentative, and rejected variables, respectively; blue represents shadow features. **(B)** Variable-importance trajectories across 100 Boruta runs. **(C)** LASSO Cox coefficient profiles. **(D)** Five-fold cross-validation for *λ* selection; dashed lines indicate lambda.min and lambda.1se. ALT: alanine aminotransferase; AST: aspartate aminotransferase; BMI: body mass index; DBP: diastolic blood pressure; eGFR: estimated glomerular filtration rate; HbA1c: glycated hemoglobin; IL-6: interleukin-6; LDL-C: low-density lipoprotein cholesterol; MLR: monocyte-to-lymphocyte ratio; PNI: prognostic nutritional index; SBP: systolic blood pressure; TC: total cholesterol; WBC: white blood cell count.

### Development, evaluation, and comparison of models

We developed and compared six machine-learning survival models to predict the risk of all-cause mortality in patients with AMI and a history of IS: a Cox proportional hazards model, an elastic-net-penalized Cox model, a RSF, a GBM survival model, an XGBoost survival model, and a survival SVM. Details of the hyperparameter settings are provided in Supplementary Table S7.

Discrimination was evaluated using C-index and time-dependent AUCs at 1, 2, and 3 years. Calibration was assessed using the IPCW Brier score, calibration intercept, calibration slope, and calibration curves. In the bootstrap OOB internal validation, the RSF achieved the highest C-index (0.720, 95% CI 0.634–0.796) but showed suboptimal calibration (calibration slope = 0.445). The elastic-net Cox model achieved a C-index comparable to that of the RSF (0.714, 95% CI 0.616–0.790), while demonstrating the best calibration (calibration slope = 0.798) and the lowest 3-year Brier score (0.096, 95% CI 0.065–0.124) (Table 2). The apparent C-index of the elastic-net Cox model was 0.732, which was close to its bootstrap-validated C-index of 0.714, suggesting limited overfitting. Pairwise comparisons of IPCW time-dependent AUCs showed no statistically significant differences at 3 years between the elastic-net Cox model and the other five models (all Holm-adjusted *P* > 0.05; Supplementary Table S8). Therefore, the elastic-net Cox model was selected as the final prediction model.

**Table 2:** Bootstrap out-of-bag internal validation performance of six survival models based on the six final predictors.

Model	Cindex	AUC_1y	AUC_2y	AUC_3y	Sensitivity_balanced	Specificity_balanced	Accuracy_balanced	F1_3y_balanced	Cal_Intercept_3y	Cal_Slope_3y	Brier_3y
RSF	0.720 (0.634-0.796)	0.703 (0.513-0.851)	0.707 (0.608-0.800)	0.712 (0.636-0.816)	0.482 (0.242-0.684)	0.784 (0.677-0.899)	0.736 (0.652-0.820)	0.359 (0.193-0.480)	-0.951	0.445	0.103
Elastic-net Cox	0.714 (0.616-0.790)	0.745 (0.629-0.843)	0.704 (0.560-0.804)	0.701 (0.601-0.801)	0.597 (0.341-0.848)	0.675 (0.530-0.822)	0.664 (0.554-0.773)	0.352 (0.213-0.488)	-0.444	0.798	0.0960
GBM	0.699 (0.614-0.774)	0.673 (0.489-0.830)	0.693 (0.578-0.797)	0.692 (0.601-0.782)	0.530 (0.290-0.735)	0.726 (0.620-0.868)	0.696 (0.624-0.796)	0.349 (0.195-0.456)	-0.879	0.516	0.101
SVM	0.696 (0.594-0.767)	0.695 (0.501-0.828)	0.700 (0.576-0.803)	0.702 (0.588-0.785)	0.561 (0.292-0.771)	0.710 (0.555-0.850)	0.687 (0.587-0.791)	0.354 (0.231-0.481)	-0.597	0.686	0.097
XGBoost	0.689 (0.606-0.760)	0.678 (0.495-0.827)	0.680 (0.567-0.776)	0.674 (0.586-0.758)	0.500 (0.228-0.730)	0.717 (0.595-0.836)	0.684 (0.606-0.757)	0.324 (0.196-0.482)	-0.961	0.497	0.104
Cox	0.688 (0.590-0.783)	0.727 (0.583-0.833)	0.668 (0.529-0.793)	0.680 (0.564-0.790)	0.580 (0.282-0.826)	0.672 (0.502-0.837)	0.657 (0.537-0.774)	0.342 (0.212-0.463)	-0.84	0.595	0.098

The unpenalized Cox reference model fitted using the same six predictors showed no evidence of a significant violation of the proportional hazards assumption, either globally or for any individual predictor (global *P* = 0.115; individual *P* values ranged from 0.065 to 0.831; Supplementary Table S9). The apparent and bootstrap-validated 1-year ROC curves for the six models are presented in Figure 3. The time-dependent AUCs of the final elastic-net Cox model at 1, 2, and 3 years were 0.745, 0.704, and 0.701, respectively (Supplementary Figure S4). Given the relatively low proportion of mortality events, IPCW time-dependent precision–recall curves were additionally generated as a supplementary assessment (Supplementary Figure S5).

**Figure 3:**
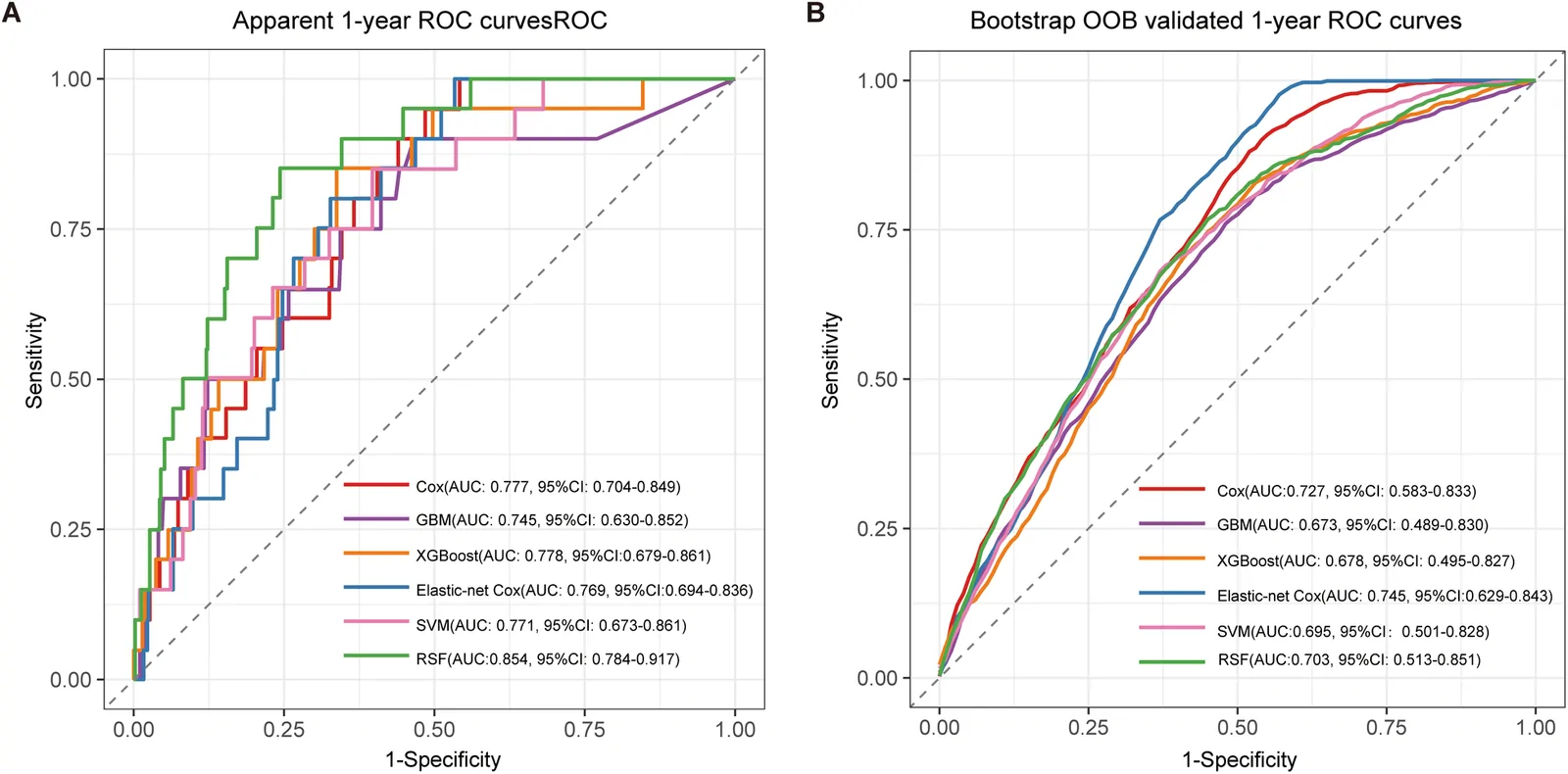
One-year time-dependent ROC curves for the six survival models. (A) Apparent performance in the development cohort. **(B)** Bootstrap out-of-bag validated performance. AUCs and 95% confidence intervals are shown in the legends. AUC: area under the curve; GBM: gradient boosting machine; OOB: out-of-bag; ROC: receiver operating characteristic; RSF: random survival forest; SVM: support vector machine.

The 3-year calibration curve showed reasonable agreement between the model-predicted risk of death and the observed mortality rate across the range of predicted risks represented in the cohort, although uncertainty was relatively large in some risk groups (Figure 4). In addition, the cohort-level mean predicted survival probability was generally consistent with the observed survival probability over follow-up (Supplementary Figure S6).

**Figure 4:**
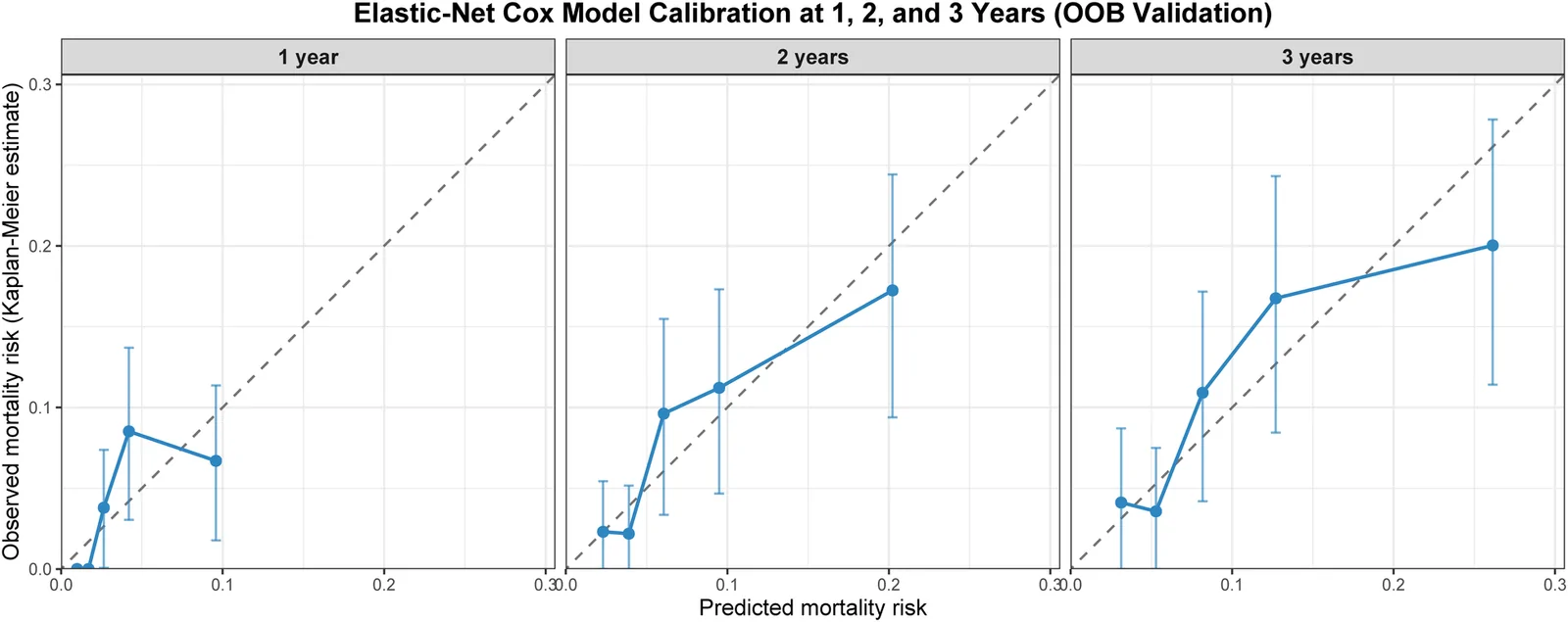
Calibration curves of the elastiC–net cox model at 1,2,and 3 years. Calibration was evaluated using bootstrap OOB predictions. Points represent observed mortality risks, error bars indicate 95% confidence intervals, and dashed lines indicate perfect calibration.

The 3-year IPCW decision curve showed that the elastic-net Cox model provided a greater net benefit than the treat-all and treat-none strategies across part of the clinically relevant threshold range, suggesting potential clinical utility (Figure 5). The model's net benefit was most evident at threshold probabilities of approximately 5%–20%, whereas its advantage diminished at higher thresholds.

**Figure 5:**
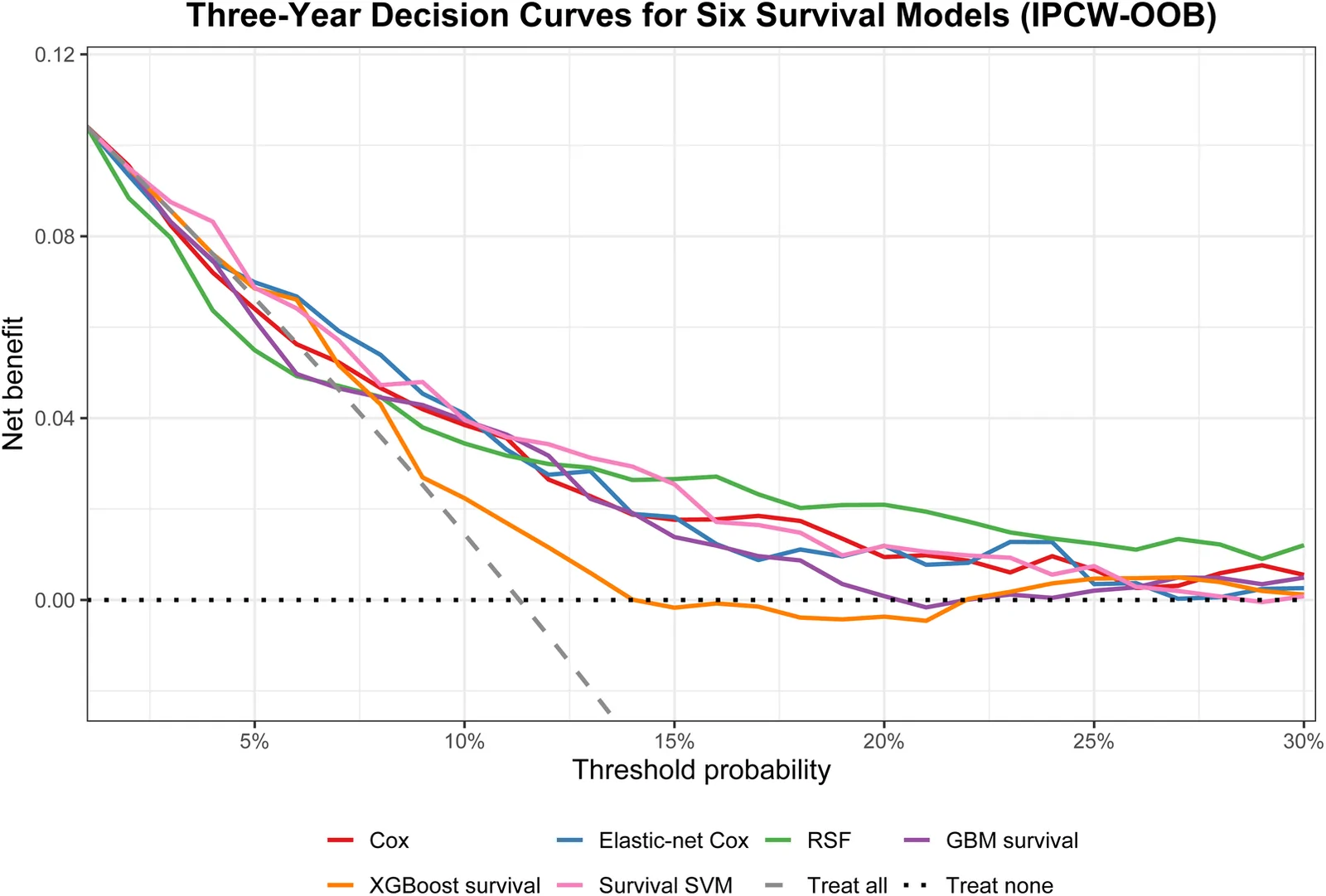
Three–year decision curves for the six survival models. Clinical net benefit across threshold probabilities was evaluated using IPCW-OOB predictions, with the treat-all and treat-none strategies as references. IPCW: inverse probability of censoring weighting.

### Model interpretation

Kernel SHAP was used to provide a global interpretation of the final elastic-net Cox model. Based on the mean absolute SHAP values, age made the largest overall contribution to model predictions, followed by hemoglobin, PNI, eGFR, AIP, and MLR (Figures 6A,B). The SHAP beeswarm plot showed that older age and higher MLR values were generally associated with a higher model-predicted risk of death, whereas higher hemoglobin, PNI, and eGFR values were associated with a lower predicted risk. Notably, higher AIP values were predominantly associated with negative SHAP values, suggesting that, within the observed data range, higher AIP generally reduced the model-predicted risk of death. This direction was consistent with the lower AIP levels observed among non-survivors at baseline and the inverse association between AIP and mortality in the univariable analysis. However, this finding is not entirely consistent with the established biological interpretation of AIP as an indicator of an atherogenic lipid profile and should therefore be interpreted cautiously.

**Figure 6:**
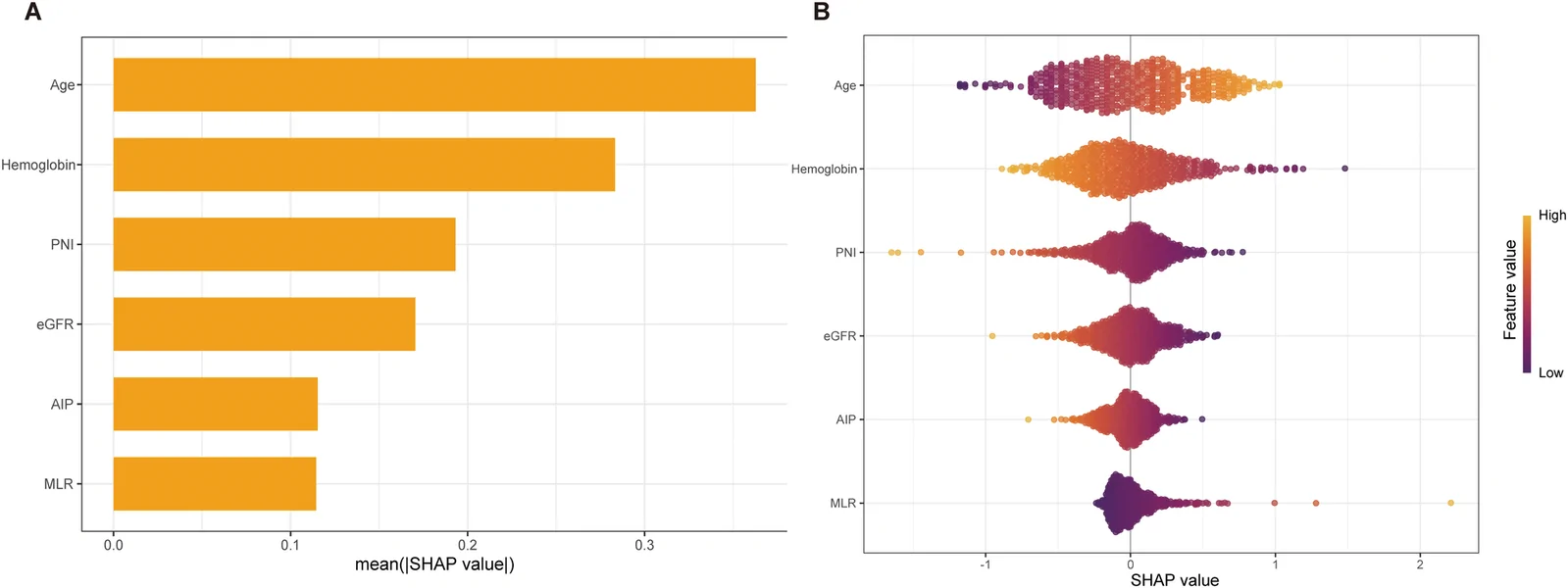
The importance of features and their contribution to the output with SHAP value evaluation. **(A)** The variable importance of different predictors in a Elastic-net Cox model. Each bar represents a predictor variable, with the length of the bar indicating its relative importance in contributing to the model's predictions. **(B)** SHAP beeswarm plot to interpret the contribution of each feature with red color increasing the interpretability while blue color decreasing the interpretability; SHAP: shapley additive explanations.

SurvSHAP(t) analysis further showed that the absolute contributions of the predictors to the predicted risk of death generally increased with the prediction horizon, whereas the relative importance ranking remained broadly stable (Supplementary Figure S7, Supplementary Figure S8). At 3 years, age and hemoglobin remained the two largest contributors, accounting for 27.6% and 24.1% of the total mean absolute SurvSHAP(t) values, respectively. They were followed by PNI (14.5%), eGFR (14.4%), MLR (11.5%), and AIP (8.0%). A similar ranking was observed at 1 year, indicating that age and hemoglobin contributed relatively consistently to model predictions across different prediction horizons.

Time-dependent permutation importance analysis further evaluated the predictive contribution of each variable from the perspective of model performance (Supplementary Figures S9A,B). At 3 years, permutation of age and hemoglobin reduced the time-dependent AUC by 0.043 and 0.041, respectively, indicating that these variables contributed most strongly to model discrimination. PNI and eGFR showed moderate contributions. When importance was measured as the increase in the Brier score after permutation, hemoglobin produced the largest permutation loss at 3 years, followed by age and eGFR, suggesting that these variables made important contributions to the accuracy of the model's predicted probabilities. The permutation losses for MLR, PNI, and AIP were comparatively small. Although AIP showed an inverse predictive attribution in the SHAP analysis, its relatively low permutation importance does not support the interpretation of AIP as an independent protective factor.

Survival partial-dependence plots further illustrated the marginal relationships between predictor values and predicted survival probabilities, with the shapes of these relationships varying across predictors (Supplementary Figure S10). Patient-level SurvSHAP(t) analysis showed that the direction and magnitude of the contributions of different predictors to the predicted risk of death varied within an individual patient and could change over the prediction horizon (Supplementary Figure S11). All these analyses were based on predictions from the final model in the development cohort and represent model-level predictive attribution rather than causal relationships or independently adjusted effects of the predictors on mortality.

### Comparison with the GRACE risk score

To evaluate the predictive value of the final elastic-net Cox model relative to an established clinical risk score, the final model was compared with the GRACE score within the same bootstrap out-of-bag internal validation framework. The final model achieved a C-index of 0.714 (95% CI 0.616–0.790), compared with 0.648 (95% CI 0.541–0.755) for the GRACE score; the paired difference was 0.065 (95% CI −0.035–0.165). The 3-year IPCW time-dependent AUC was 0.701 (95% CI 0.601–0.801) for the final model and 0.608 (95% CI 0.477–0.723) for the GRACE score, with a paired difference of 0.093 (95% CI −0.031–0.234) (Supplementary Table S10). Therefore, the final model could not be considered statistically superior to the GRACE score in terms of discrimination.

The 3-year calibration intercept and slope were −0.132 and 0.931, respectively, for the final model, compared with −0.651 and 0.676 for the GRACE score, suggesting better agreement between predicted probabilities and observed risks for the final model (Supplementary Figure S12). The 3-year decision curve showed that, across part of the clinically relevant threshold range, the final model yielded a higher net benefit than the GRACE score and the treat-all and treat-none strategies, suggesting potentially greater clinical utility (Supplementary Figure S13). However, the calibration and decision-curve comparisons were conducted in the development cohort and require further validation in an independent external cohort.

In the exploratory incremental-value analysis, adding the final-model risk score to the GRACE score yielded a 3-year continuous NRI of 0.558 (95% CI 0.189–0.839) and an IDI of 0.049 (95% CI 0.013–0.119) (Supplementary Table S11). Because this analysis was based on models fitted using the complete development cohort, these findings also require confirmation in an independent external cohort. Given that the GRACE score was originally developed primarily for shorter-term prognostic assessment, we additionally compared the 6-month predictive performance of the two models; the results are presented in Supplementary Table S12 and Supplementary Figure S14.

## Discussion

In this study, we developed and internally validated six survival prediction models：Cox, elastic-net Cox, RSF, GBM survival, XGBoost survival, and survival SVM—for predicting all-cause mortality in patients with AMI and prior IS. Although the RSF achieved the numerically highest C-index in bootstrap OOB validation, the elastic-net Cox model demonstrated comparable discrimination, better calibration, a lower 3-year Brier score, and greater clinical interpretability, and was therefore selected as the final model. The elastic-net Cox model yielded a bootstrap OOB C-index of 0.714, with 1-, 2-, and 3-year IPCW time-dependent AUCs of 0.745, 0.704, and 0.701, respectively. Model interpretation analyses identified age and hemoglobin as the largest contributors to model predictions. Compared with the GRACE score, the elastic-net Cox model showed numerically better discrimination and calibration; however, the difference in discrimination was not statistically significant.

Machine learning models have demonstrated robust prognostic utility in patients with AMI or IS alone (12, 28). However, their application has largely been confined to single-disease cohorts, leaving the comorbid population with concurrent AMI and IS inadequately addressed. This gap is clinically consequential because the two conditions are pathophysiologically coupled through shared substrates—atherosclerosis, systemic inflammation, and endothelial dysfunction—and bidirectional brain–heart axis crosstalk (29). Rather than representing isolated vascular events, AMI and IS potentiate one another via autonomic, inflammatory, and hemodynamic pathways, generating a mortality risk that exceeds the simple sum of individual disease effects. Consequently, developing a dedicated risk prediction framework for this high-risk comorbid population is clinically imperative and scientifically warranted.

In the present study, hemoglobin and PNI made important contributions to mortality-risk predictions generated by the final elastic-net Cox model. PNI, which is calculated from serum albumin and lymphocyte count, is commonly regarded as a composite indicator of nutritional reserve and immune status. A lower PNI may reflect malnutrition, systemic inflammation, and impaired immune function, thereby indicating reduced physiological reserve and diminished tissue-repair capacity following acute ischemic injury (30). Lower hemoglobin also generally shifted model predictions toward higher mortality risk, possibly because of reduced oxygen-carrying capacity and inadequate oxygen delivery to myocardial and cerebral tissues. In addition, reduced hemoglobin in this population may be associated with chronic inflammation, renal dysfunction, bleeding, or other comorbidities rather than nutritional deficiency alone (31) Although PNI and hemoglobin reflect different clinical dimensions, their joint contributions to model predictions may capture an overall state of reduced physiological reserve and increased vulnerability to ischemic injury. This interpretation is consistent with previous evidence linking poor nutritional and hematological status to adverse cerebrocardiovascular outcomes (32, 33).

MLR also contributed to model predictions, with higher MLR values generally shifting predictions toward greater mortality risk. As a composite inflammatory marker, MLR may reflect the balance between monocyte-mediated innate immune activation and lymphocyte-related adaptive immunity (34). Given the involvement of chronic inflammation in endothelial dysfunction, atherosclerotic plaque progression, and plaque instability, this predictive direction has some biological plausibility. However, because the present study focused on prediction modeling, these findings do not establish that MLR independently or causally influences mortality.

The predictive direction of AIP differed from that reported in some previous studies and therefore requires cautious interpretation. Although AIP is commonly considered an indicator of an atherogenic lipid profile and insulin resistance (35), higher AIP values in the present study predominantly shifted model predictions toward lower mortality risk. This inverse predictive attribution was consistent with the baseline comparison and univariable analysis but was not fully consistent with the conventional biological interpretation of AIP. Notably, previous evidence suggests that the relationship between AIP and clinical outcomes may not be a simple positive linear association. A study of patients with stroke found that higher AIP was associated with lower all-cause mortality, demonstrating a so-called “lipid paradox” (36). This finding provides some context for the inverse predictive pattern observed in our study and suggests that the prognostic significance of AIP may vary according to the study population, outcome definition, and range of AIP values. Acute-phase changes in lipid concentrations, previous or in-hospital lipid-lowering treatment, nutritional status, residual confounding, selection or survivor bias, and correlations among predictors may have influenced our findings. Moreover, AIP showed relatively limited importance in the permutation analysis. Therefore, this inverse predictive attribution should not be interpreted as evidence that higher AIP has an independent protective effect. The potentially complex or nonlinear relationship between AIP and mortality risk requires further investigation in larger independent cohorts.

The prognostic significance of renal impairment has been well established in isolated cardiovascular and cerebrovascular diseases. Luo et al. (37) demonstrated that even mild reductions in eGFR independently predict 1-year mortality in patients with STEMI, whereas Zhang et al. (38) showed that creatinine–cystatin C-based eGFR predicts long-term adverse outcomes after ischemic stroke. These studies establish eGFR as an important prognostic indicator across distinct vascular conditions. However, its prognostic role in patients with concurrent acute myocardial infarction and ischemic stroke remains poorly defined. The present study addresses this gap by confirming eGFR as an independent predictor of all-cause mortality in a cohort of patients with acute myocardial infarction and prior stroke. Pathophysiologically, renal insufficiency may exacerbate systemic vascular injury through interconnected pathways. Reduced eGFR promotes the accumulation of uremic toxins, which activate nuclear factor-kappa B and stimulate the release of interleukin-6 and tumor necrosis factor-alpha. Impaired renal clearance and diminished antioxidant capacity facilitate excessive reactive oxygen species generation, leading to endothelial dysfunction, lipid peroxidation, and atherosclerotic plaque destabilization. Furthermore, activation of the RAAS increases angiotensin II levels, which promote vascular smooth muscle cell proliferation, collagen deposition, and myocardial fibrosis, thereby accelerating systemic arteriosclerosis (39–41). These mechanisms are supported by the INTERASPIRE multinational study, in which Al-Azzawy et al. (42) identified CKD as a strong yet frequently underrecognized risk factor for coronary artery disease, demonstrating a significant association with the composite endpoints of cardiovascular death, recurrent myocardial infarction, and stroke. Collectively, these findings indicate that eGFR functions not only as a marker of renal function but also as a comprehensive indicator of systemic vascular vulnerability and adverse outcomes in patients with concurrent cardiocerebral infarction events.

The predictive validity of machine learning algorithms is contingent, in part, upon data quality and sufficient sample size (43). In the present study, three distinct feature selection approaches—Boruta algorithm, LASSO regression, and univariate Cox regression—were employed, and the six variables jointly identified by all three methods were used as a pre-specified predictor set for model development. This consistency indicates that the identified variable set exhibits robustness and reproducibility across methodologically disparate strategies, thereby providing a reliable feature basis for model development. Furthermore, machine learning algorithms are frequently constrained by the “black box” limitation, wherein the mechanistic underpinnings of specific outputs remain intractable (44). Recently, explainable machine learning frameworks have been advanced to reconcile the predictive performance of black box models with output interpretability, among which SHAP has emerged as the most widely adopted analytical methodology (45). By quantifying the marginal contribution of individual features to model predictions, SHAP enhances model transparency, interpretability, and reliability. Additionally, SHAP values enable a precise and stable assessment of relative feature importance, offering methodological advantages over conventional feature evaluation approaches.

### Limitations

Several limitations should be acknowledged. First, this prospective single-center study had a modest sample size and a limited number of outcome events. Only 66 deaths occurred among 543 patients, corresponding to an event rate of 12.2%. Although the six-variable model had approximately 11 events per variable, the limited absolute number of events reduced the effective information available for model development, contributed to wide confidence intervals for some performance estimates, and increased the risk of overfitting, particularly for flexible machine-learning algorithms. Second, internal validation was performed using 100 bootstrap resamples with out-of-bag evaluation. Although this approach reduces optimism in performance estimates, bootstrap resampling does not generate new independent information, increase the effective sample size, or replace validation in an independent population. Third, stroke severity and neurological status were not directly evaluated using measures such as the NIHSS or mRS. Information on infarct severity, treatment changes, medication adherence, rehabilitation, and post-discharge management was also incomplete. These factors may provide additional prognostic information and improve model performance. Fourth, despite the inclusion of multiple clinical and laboratory variables, unmeasured or incompletely measured factors may have influenced the observed associations and model predictions. Finally, the model has not undergone prospective, real-time clinical implementation testing. Independent temporal and geographical validation, together with prospective assessment of its feasibility, clinical acceptability, integration into routine workflows, and impact on clinical decisions and patient outcomes, is required before clinical application.

## Conclusion

we developed and internally validated a survival-based machine-learning model incorporating six clinical variables to predict post-discharge mortality following PCI in patients with acute myocardial infarction and prior ischemic stroke. The elastic-net Cox model provided the most favorable overall balance of discrimination, calibration, prediction error, and clinical interpretability. Although the elastic-net Cox model showed numerically better performance than the established GRACE risk score in the development cohort, statistical superiority was not demonstrated. Age and hemoglobin had the largest contributions to model predictions, whereas PNI, eGFR, AIP, and MLR provided additional predictive information. The model may help clinicians identify patients at high risk of adverse outcomes and inform follow-up intensity and secondary prevention strategies. Future multicenter studies and independent external validation are required to confirm and extend these findings.

## Data Availability

The raw data supporting the conclusions of this article will be made available by the authors, without undue reservation.
