# Supplementary material for: Mortality prediction in patients with acute myocardial infarction and prior ischemic stroke: a prospective cohort study with explainable machine learning and SHAP analysis
Source: Front Cardiovasc Med. 2026 Oct 27;13:1949226. doi: 10.3389/fcvm.2026.1949226 (PMC13748428; doi:10.3389/fcvm.2026.1949226)

Supplemental Material

Mortality Prediction in Patients With Acute Myocardial Infarction and Prior Ischemic Stroke: A Prospective Cohort Study With Explainable Machine Learning and SHAP Analysis

Congcong Han, Abudureheman Muladili, Ning Song, Chunfang Shan, Fen Liu, Xiaomei Li, Qian Zhao, Yining Yan

Methods

Definition of AMI subtypes

All AMI patients (ST-elevation myocardial infarction [STEMI] and non-ST elevation myocardial infarction [NSTEMI]) recruited in this study were adjudicated by 3 experienced cardiologists by the latest guideline at recruitment.

Both STEMI and NSTEMI were diagnosed in accordance with the Third Universal Definition of Myocardial Infarction (2012) and the Fourth Universal Definition of Myocardial Infarction (2018)[1,2]. More specifically, STEMI was adjudicated as having chest pain or other ischemic symptoms that develop ST elevation at the J point in two contiguous leads with the cut-points: ≥0.1 mV in all leads other than leads V2-V3 where the following cut-points applied: ≥0.2 mV in men ≥40 years; ≥0.25 mV in men <40 years, or ≥0.15 mV in women. In contrast, NSTEMI was adjudicated as having chest pain or other ischemic symptoms with ST depression ≥0.05 mV in two contiguous leads and/or T wave inversion ≥0.1 mV in two contiguous leads with prominent R wave or R/S ratio >1.

Reference

1. Thygesen, K. et al. Third universal definition of myocardial infarction. *Circulation* **126**, 2020-2035 (2012).

2. Thygesen, K. et al. Fourth universal definition of myocardial infarction (2018). *European heart journal* **40**, 237-269 (2019).

Supplementary Table 2. Proportion of missing values for each candidate predictor

Variable	N_missing	Pct_missing
BMI	76	14.00
Heartrate	71	13.08
IL-6	52	9.58
HbA1c	27	4.97
Procalcitonin	21	3.87
TG	10	1.84
TC	10	1.84
HDL-C	10	1.84
LDL-C	10	1.84
D-Dimer	8	1.47
WBC	6	1.10
Total Bilirubin	4	0.74
Age	0	0
Smoking	0	0
RDW	0	0
MCV	0	0
Neutrophil percentage	0	0
Eosinophil count	0	0
Hemoglobin	0	0
Urea	0	0
Creatinine	0	0
RBC	0	0
Direct Bilirubin	0	0
Total Protein	0	0
ALT	0	0
Hematocrit	0	0
eGFR	0	0
SBP	0	0
DBP	0	0
Hypertension	0	0
Diabetes	0	0
Platelet count	0	0
AST	0	0
Gender	0	0
Albumin	0	0
Lymphocyte count	0	0
Monocyte count	0	0

NT-ProBNP: n-terminal pro-B-type natriuretic peptide; LVEF: left ventricular ejection fraction; BMI: body mass index; IL-6: interleukin-6; HbA1c:haemoglobin A1c; TC: total cholesterol; TG: triglyceride; HDL-C: high-density lipoprotein cholesterol; LDL-C: low-density lipoprotein cholesterol; WBC: white blood cell count; RDW: red blood cell distribution width; MCV: mean corpuscular volume; RBC: red blood cell; ALT: alanine aminotransferase; eGFR: estimated

glomerular filtration rate; SBP: systolic blood pressure; DBP: diastolic blood pressure; AST: Aspartate Aminotransferase;

Supplementary Table 2. Univariate Cox analysis to screen the features.

Variable	HR	CI_lower	CI_upper	P_value
Age	1.063	1.04	1.085	<0.001
Hemoglobin	0.965	0.951	0.978	<0.001
HbA1c	1.842	1.418	2.394	<0.001
Hematocrit	0.894	0.85	0.94	<0.001
PNI	0.905	0.865	0.947	<0.001
BMI	0.866	0.791	0.949	0.002
eGFR	0.988	0.98	0.996	0.003
AIP	0.346	0.155	0.773	0.010
MLR	3.247	1.264	8.341	0.014
DBP	0.979	0.961	0.997	0.022
Neutrophil percentage	1.024	1.001	1.046	0.036
Procalcitonin	169711.959	1.182	24366781514	0.047
TC	0.821	0.65	1.038	0.099
LDL-C	0.823	0.634	1.068	0.143
Platelet count	0.997	0.993	1.001	0.162
Total Bilirubin	1.032	0.984	1.081	0.194
Diabetes	1.386	0.839	2.289	0.203
Gender	1.389	0.78	2.472	0.264
IL-6	1.022	0.983	1.063	0.270
ALT	0.992	0.975	1.009	0.351
Hypertension	1.203	0.731	1.982	0.467
WBC	1.027	0.953	1.106	0.488
Total Protein	0.988	0.95	1.028	0.556
SBP	0.997	0.985	1.009	0.592
heartrate	1.002	0.985	1.019	0.843
Direct Bilirubin	1.011	0.813	1.258	0.920
AST	1	0.993	1.007	0.932

PNI: prognostic nutritional index; AIP: atherogenic index of plasma; MLR: monocyte-to-lymphocyte ratio

Supplementary Table 3. Results of Boruta Feature Selection and Variable Importance Statistics

Variable	meanImp	medianImp	minImp	maxImp	normHits	decision
HbA1c	2.146849486	2.147930229	-0.345305812	4.78043127	0.474747475	Tentative
Gender	1.333707348	1.425783524	-0.447714073	3.192142653	0.090909091	Rejected
Total Bilirubin	-0.435053	-0.595465	-1.542153	1.843718	0.010101	Rejecte

	248	237	155	045	01	d
LDL-C	0.937482001	1.042958864	-0.616207274	1.970663421	0	Rejected
TC	1.484108187	1.662431547	-0.335265998	3.90383251	0.111111111	Rejected
AST	2.329476372	2.288680319	0.06578412	5.110619734	0.464646465	Tentative
Platelet count	-0.40004299	-0.556805585	-1.911801097	1.725143571	0	Rejected
Diabetes	-0.394000329	0.244293883	-2.67792342	1.451904443	0	Rejected
Hypertension	0.053318107	-0.026959365	-0.576758416	0.834324351	0	Rejected
heartrate	-0.053724126	-0.355496085	-1.876586495	2.831873575	0.01010101	Rejected
DBP	0.917214928	1.307029245	-0.443405761	2.206668162	0	Rejected
SBP	0.059507524	-0.181439156	-2.085029396	2.15971442	0.01010101	Rejected
eGFR	3.289868512	3.293086597	0.179166455	6.556398704	0.737373737	Confirmed
MLR	6.127289568	6.078586031	3.768395508	9.135431673	0.96969697	Confirmed
AIP	3.300559883	3.283354044	1.010386654	6.521714379	0.747474747	Confirmed
PNI	6.931495551	7.078150373	3.600881379	9.10286539	1	Confirmed
WBC	4.870605431	4.76910229	1.530982852	7.611624611	0.919191919	Confirmed
BMI	1.268958879	1.21619724	-1.167864987	3.7663764	0.161616162	Rejected
Procalcitonin	3.654498071	3.717692642	0.807530952	6.218679485	0.868686869	Confirmed
IL-6	-0.085729237	-0.336932056	-2.1017454	1.503427578	0	Rejected
Hematocrit	6.34277733	6.404594985	2.267757234	9.289113755	0.95959596	Confirmed
ALT	3.371194194	3.455193842	0.109722795	5.713448623	0.787878788	Confirmed
Total Protein	2.389836211	2.375594369	-0.029900836	6.102705214	0.535353535	Tentative
Direct Bilirubin	0.838573578	1.011573105	-0.927229956	3.03990165	0.090909091	Rejected
Hemoglobin	8.1817106	8.2956397	4.7506673	11.20713	1	Confirm

	04	68	4	343		ed
Neutrophil percentage	0.939109184	1.014800433	-1.314403493	2.731653842	0	Rejected
Age	13.25864231	13.32053013	9.96151928	17.22744386	1	Confirmed

Supplementary Table 4. Predictor Coefficients and Feature-Selection Results from LASSO Cox Regression

Variable	Coef_lambda_min	Coef_lambda_1se	Selected_min	Selected_1se
Age	0.0343	0	yes	no
Neutrophil percentage	0	0	no	no
Hemoglobin	-0.0172	0	yes	no
Direct Bilirubin	0	0	no	no
Total Protein	0	0	no	no
ALT	0	0	no	no
Hematocrit	0	0	no	no
IL-6	0	0	no	no
Procalcitonin	0	0	no	no
BMI	-0.0336	0	yes	no
WBC	0.0597	0	yes	no
PNI	-0.0272	0	yes	no
AIP	-0.1331	0	yes	no
MLR	0.0818	0	yes	no
eGFR	-9.00E-04	0	yes	no
SBP	0	0	no	no
DBP	0	0	yes	no
Heartrate	0	0	no	no
Hypertension	0	0	no	no
Diabetes	0	0	no	no
Platelet count	0	0	no	no
AST	0	0	no	no
TC	0	0	no	no
LDL-C	-0.0589	0	yes	no
Total Bilirubin	6e-04	0	yes	no
Gender	-0.018	0	yes	no
HbA1c	0.3561	0	yes	no

Supplementary Table 5. Summary of Results from the Three Feature-Selection Methods

Variable	Cox	Boruta	LASSO	N
Age	·	·	·	3
AIP	·	·	·	3
MLR	·	·	·	3

PNI	·	·	·	3
eGFR	·	·	·	3
Hemoglobin	·	·	·	3
BMI	·		·	2
DBP	·		·	2
WBC		·	·	2
Hematocrit	·	·		2
Procalcitonin	·	·		2
HbA1c	·		·	2
LDL-C			·	1
Gender			·	1
TC	·			1
ALT		·		1
Neutrophil percentage	·			1
Total Bilirubin			·	1

Supplementary Table 6. Variance Inflation Factors for the Six Final Predictors

Variable	VIF
PNI	1.52
Hemoglobin	1.38
MLR	1.27
Age	1.21
eGFR	1.2
AIP	1.13

Supplementary Table 7. Hyperparameter Settings of the Six Tuned Survival Models

Model	Key Hyperparameters
Cox	-
Elastic-net Cox	alpha: 0, 0.25, 0.50, 0.75, 1.00; selected: 0.00<br>lambda: selected by 5-fold cross-validation using lambda.1se<br>standardization: TRUE
RSF	mtry: 2, 3, 5; selected: 2<br>nodesize: 20, 30, 40; selected: 30<br>ntree: 300<br>nsplit: 5
GBM survival	interaction.depth: 1, 2; selected: 1<br>shrinkage: 0.01, 0.03; selected: 0.03<br>n.trees: maximum 600<br>n.minobsinnode: 20<br>optimal number of trees: selected using OOB error
XGBoost survival	objective: survival:cox<br>max_depth: 1, 2; selected: 1

	eta: 0.01, 0.03; selected: 0.03<br>nrounds: 100, 200; selected: 200<br>subsample: 0.70<br>colsample_bytree: 0.80<br>min_child_weight: 10<br>lambda: 10<br>alpha: 0.1<br>early stopping: not applied
Survival SVM	type: regression<br>kernel: radial basis function (RBF)<br>gamma.mu: 0.1, 1; selected: 0.1<br>sigma²: 5, 10; selected: 5<br>solver: quadprog; ipop used as fallback<br>class weighting: not applied

Supplementary Table 8. Pairwise Comparisons of IPCW Time-Dependent AUCs Between the Final Elastic-Net Cox Model and the Other Models at 1, 2, and 3 Years

Time	Comparison	AUC_Selected	AUC_Comparator	AUC_Diff	CI_lower	CI_upper	P_boot	N_Paired	P_Holm
1 year	Elastic-net Cox vs Cox	0.7447	0.7275	0.0172	-0.0421	0.0776	0.673	100	1
1 year	Elastic-net Cox vs RSF	0.7447	0.7027	0.042	-0.0579	0.1502	0.455	100	1
1 year	Elastic-net Cox vs GBM	0.7447	0.6733	0.0713	-0.0317	0.2016	0.178	100	1
1 year	Elastic-net Cox vs XGBoost	0.7447	0.6779	0.0667	-0.0308	0.1766	0.158	100	1
1 year	Elastic-net Cox vs SVM	0.7447	0.6948	0.0499	-0.029	0.1698	0.376	100	1
2 year	Elastic-net Cox vs Cox	0.7035	0.6682	0.0354	-0.0183	0.1071	0.297	100	1
2 year	Elastic-net Cox vs RSF	0.7035	0.7068	-0.0032	-0.1035	0.0848	1	100	1
2 year	Elastic-net Cox vs GBM	0.7035	0.6928	0.0107	-0.0767	0.0941	0.752	100	1
2 year	Elastic-net Cox vs XGBoost	0.7035	0.6799	0.0237	-0.0463	0.099	0.535	100	1
2 year	Elastic-net Cox vs SVM	0.7035	0.7002	0.0033	-0.0699	0.1	0.95	100	1
3 year	Elastic-net Cox vs Cox	0.7009	0.6796	0.0213	-0.0262	0.0879	0.515	100	1
3 year	Elastic-net Cox vs RSF	0.7009	0.7123	-0.0115	-0.0784	0.0574	0.752	100	1

3 year	Elastic-net Cox vs GBM	0.7009	0.6924	0.0085	-0.0656	0.0816	0.851	100	1
3 year	Elastic-net Cox vs XGBoost	0.7009	0.6739	0.027	-0.036	0.0999	0.475	100	1
3 year	Elastic-net Cox vs SVM	0.7009	0.7024	-0.0015	-0.0675	0.0942	0.871	100	1

Supplementary Table 9. Assessment of the Cox Proportional Hazards Assumption Using Schoenfeld Residuals

Variable	chisq	df	*P*
Age	3.405417079	1	0.064982685
Hemoglobin	1.164905475	1	0.280450506
PNI	3.393052705	1	0.065471628
MLR	0.045717223	1	0.830690655
eGFR	0.142667911	1	0.705643022
AIP	0.797103416	1	0.371960814
GLOBAL	10.22640856	6	0.115435592

Supplementary Table 10. Comparison of discrimination between the final elastic-net Cox model and the GRACE score in bootstrap OOB internal validation

Metric	Estimate
Bootstrap OOB C-index(Elastic-net Cox)	0.714 (0.616-0.790)
Bootstrap OOB C-index (GRACE)	0.648 (0.541-0.755)
difference(Elastic-net Cox-GRACE)	0.065 (-0.035-0.165)
Bootstrap OOB 3-years AUC(Elastic-net Cox)	0.701 (0.601-0.801)
Bootstrap OOB 3-years AUC(GRACE)	0.608 (0.477-0.723)
difference(Elastic-net Cox-GRACE)	0.093 (-0.031-0.234)

Supplementary Table 11. Exploratory incremental predictive value of the final elastic-net Cox model over the GRACE score at 3 years

**Measure**	**Estimate**	**CI_lower**	**CI_upper**	**N**	**Time_year**
IDI	0.049307761	0.013486626	0.118742048	543	3
Continuous_NRI_gt0	0.557706237	0.189310133	0.839175195	543	3
Median_risk_improvement	0.04002001	0.010505253	0.089544772	543	3
Added_risk_score_P	1.21E-07	NA	NA	543	NA

Supplementary Table 12. Six-month discrimination performance of the final elastic-net Cox model and the GRACE score

Metric	Value	P_value
Bootstrap OOB C-index(Elastic-net Cox)	0.714 (0.616-0.790)	

Bootstrap OOB C-index(GRACE)	0.648 (0.541-0.755)	
Bootstrap OOB 3 年 AUC(Elastic-net Cox)	0.701 (0.601-0.801)	
Bootstrap OOB 3 年 AUC(GRACE)	0.608 (0.477-0.723)	
Apparent C-index (full follow-up)(Elastic-net Cox)	0.732	
Apparent C-index (full follow-up)(GRACE)	0.645	
Apparent 3-year AUC(Elastic-net Cox)	0.721	
Apparent 3-year AUC(GRACE)	0.606	
Apparent 6-year AUC(Elastic-net Cox)	0.743	
Apparent 6-year AUC(GRACE)	0.702	
3-year calibration intercept(Elastic-net Cox)	-0.132	
3-year calibration intercept(GRACE)	-0.651	
3-year calibration slope(Elastic-net Cox)	0.931	
3-year calibration slope(GRACE)	0.676	
C-index difference(Elastic-net Cox-GRACE)	0.0905(0.0204-0.1614)	0.024
Difference in 3-year IPCW AUC(Elastic-net Cox-GRACE)	0.1156(0.0254-0.2135)	0.012
Difference in 6-year IPCW AUC(Elastic-net Cox-GRACE)	0.048(-0.1235-0.2402)	0.599

Supplementary Figure 1. Trace plots for convergence assessment of the multiple imputation chains

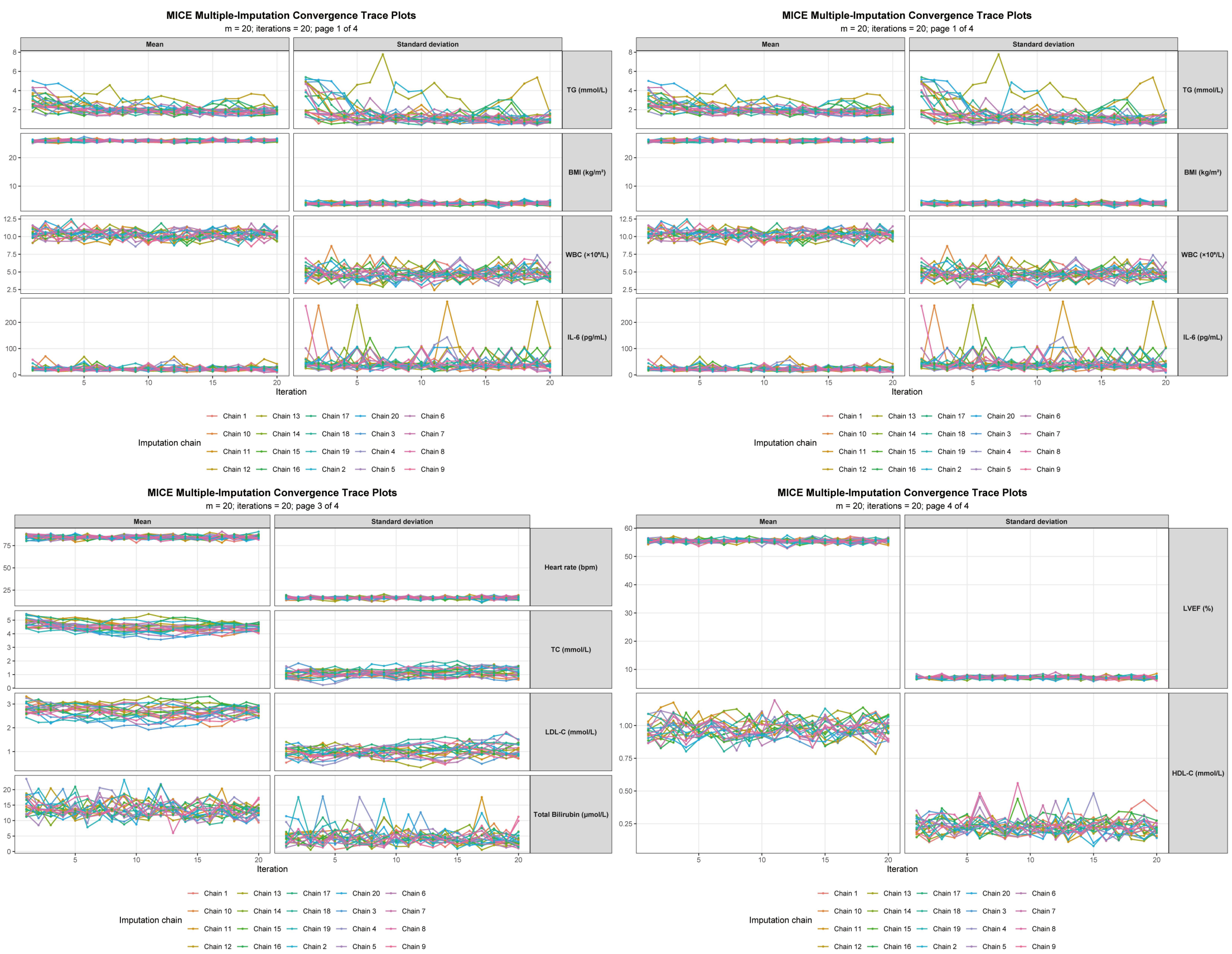

Supplementary Figure 2. Venn Diagram of Features Selected by Three Feature-Selection Methods

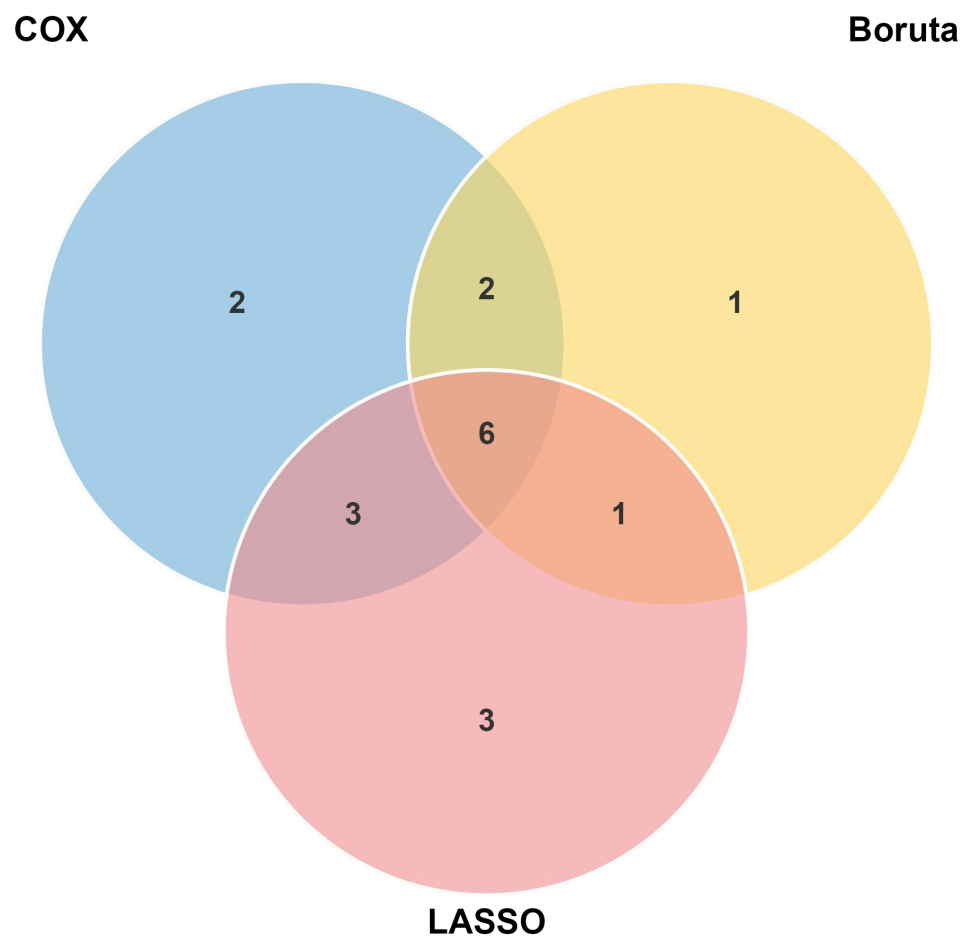

Supplementary Figure 3. Multicollinearity Diagnostics for the Final Predictors

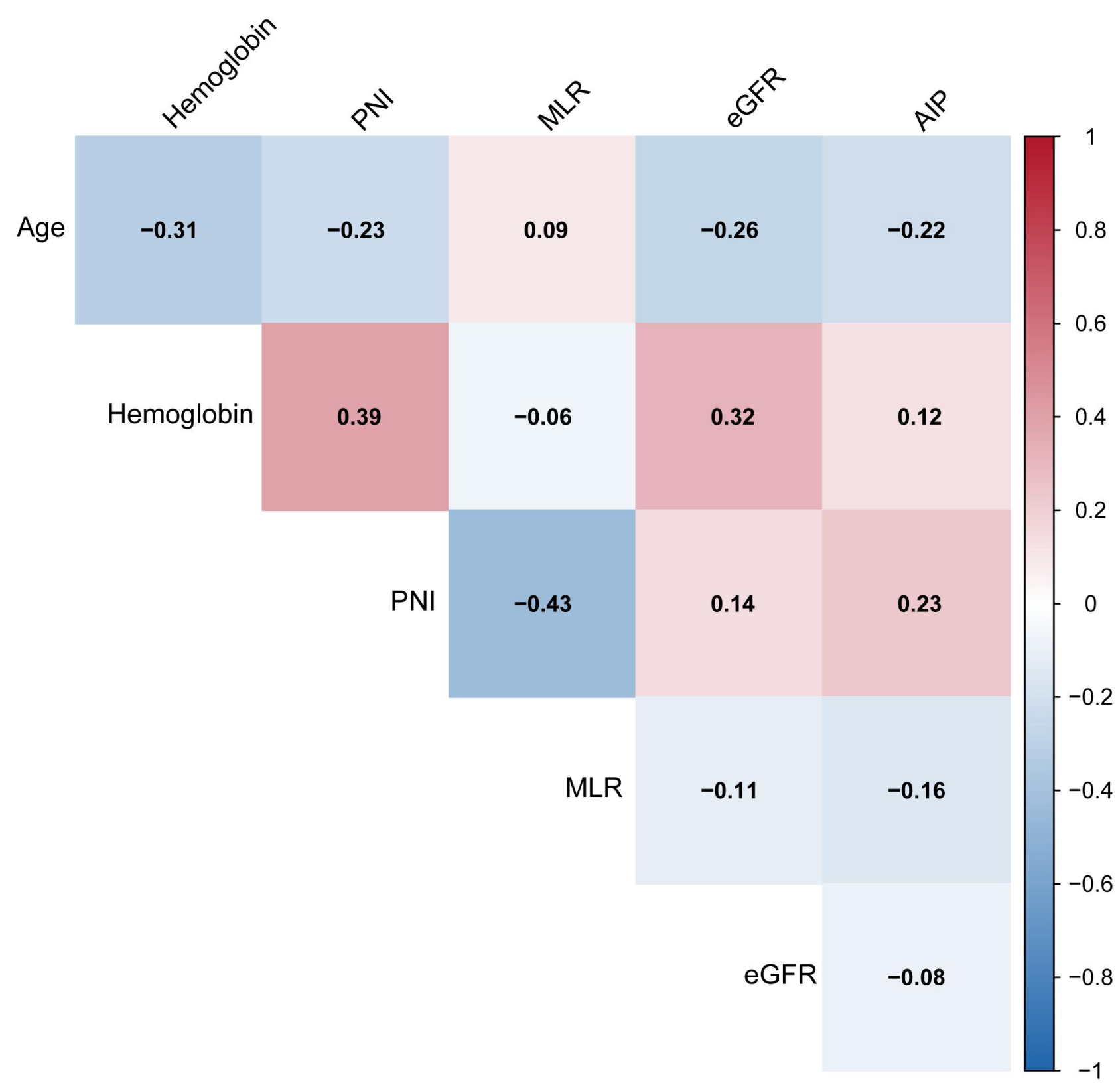

Supplementary Figure 4. Bootstrap out-of-bag (OOB) validated 1-, 2-, and 3-year ROC curves of the final elastic-net Cox model. A Bootstrap OOB validated 1-year ROC. B Bootstrap OOB validated 2-year ROC. C.Bootstrap OOB validated 3-year ROC

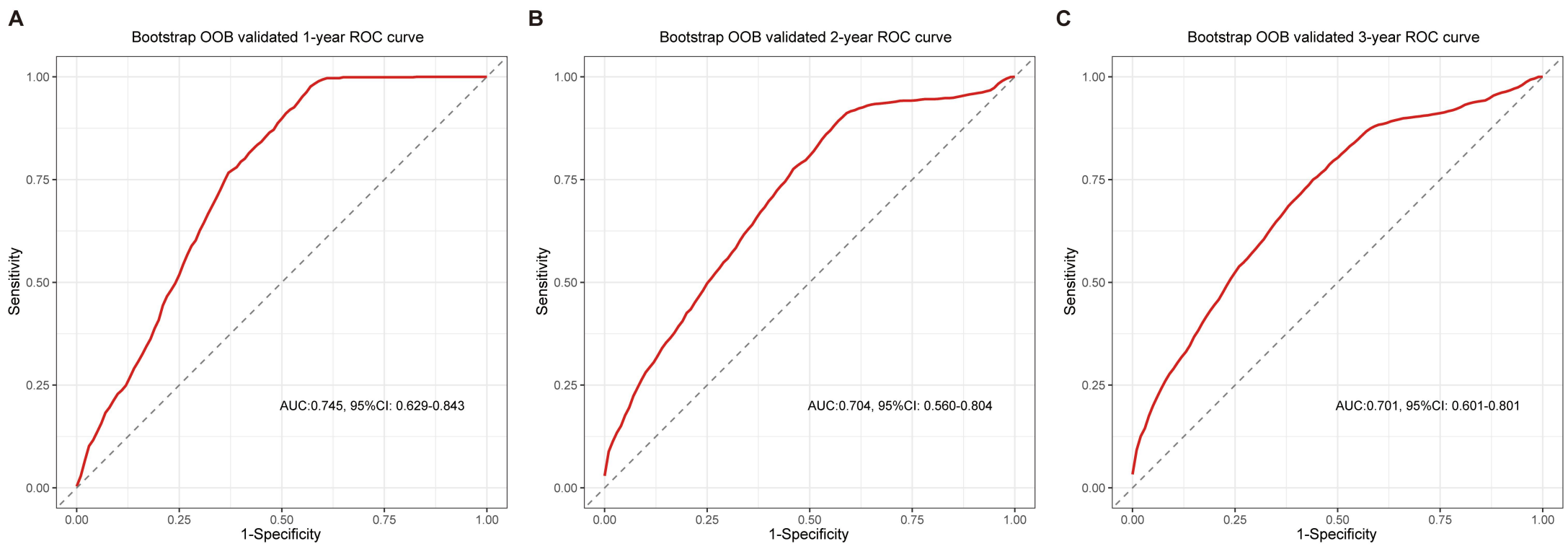

Supplementary Figure 5. 3-year precision-recall (PR) curves of the six survival prediction models (IPCW).

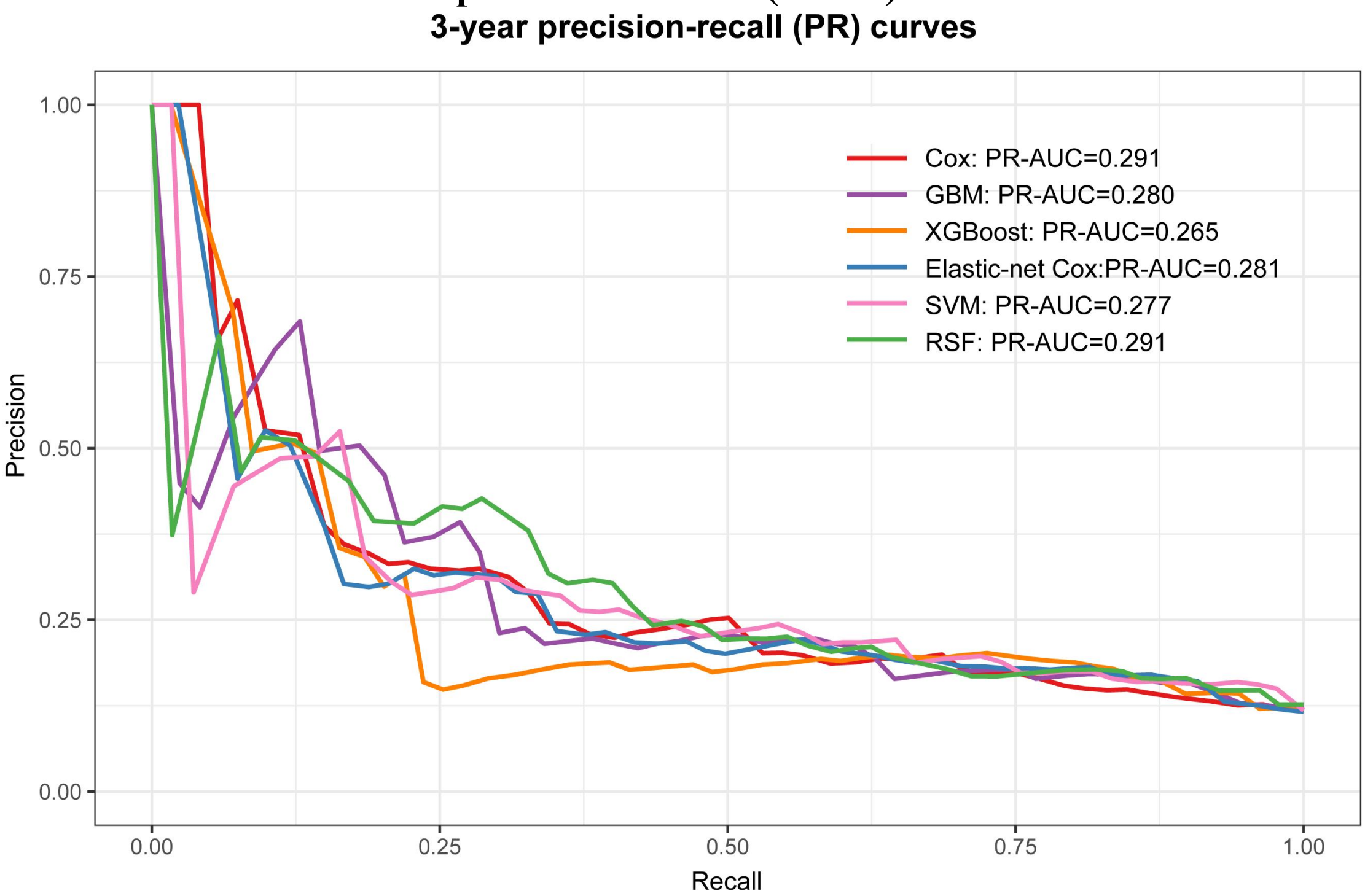

Supplementary Figure 6. Time-dependent calibration of the final model over 0–3 years.

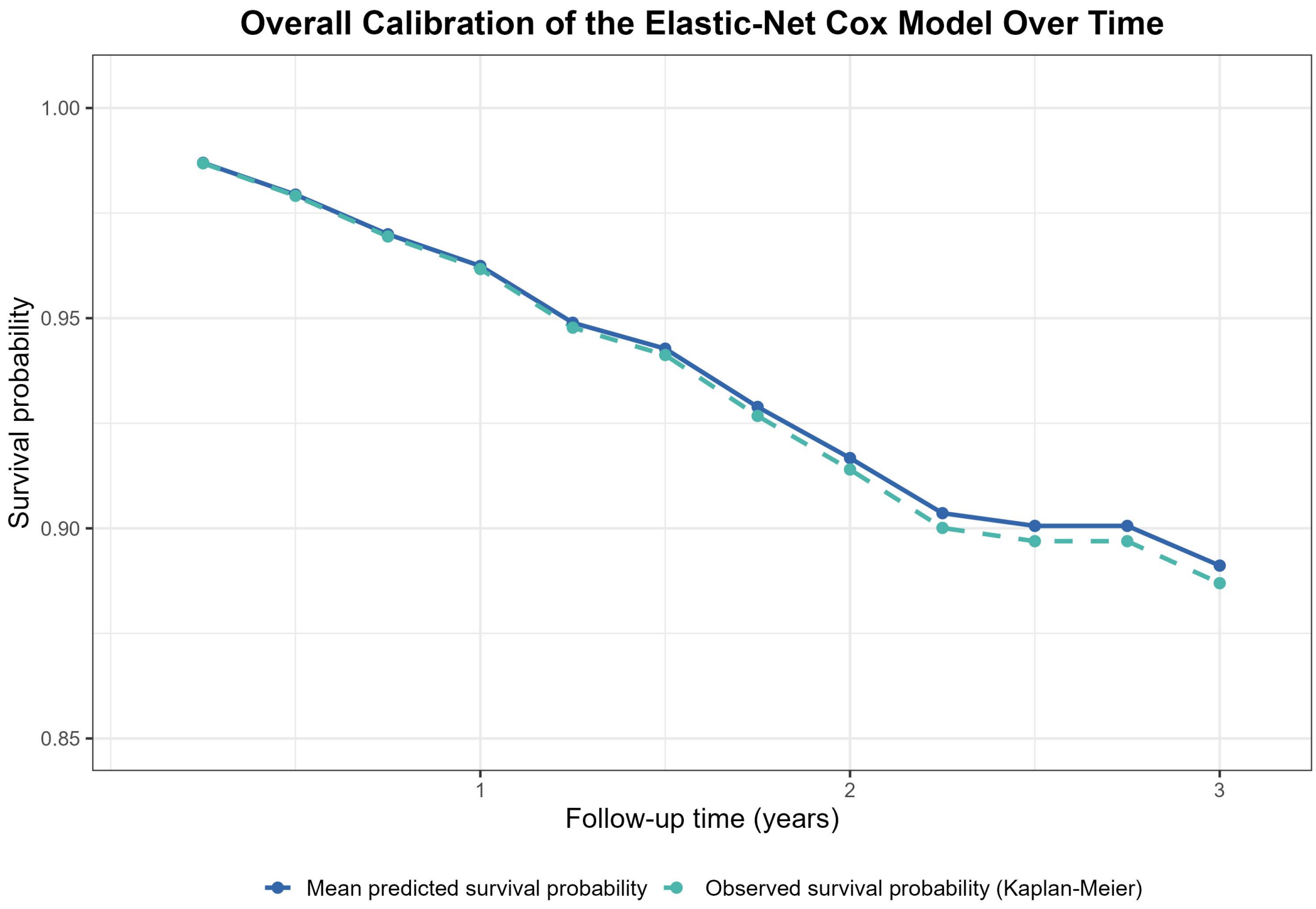

Supplementary Figure 7. Time-dependent calibration of the final model over 0–3 years.

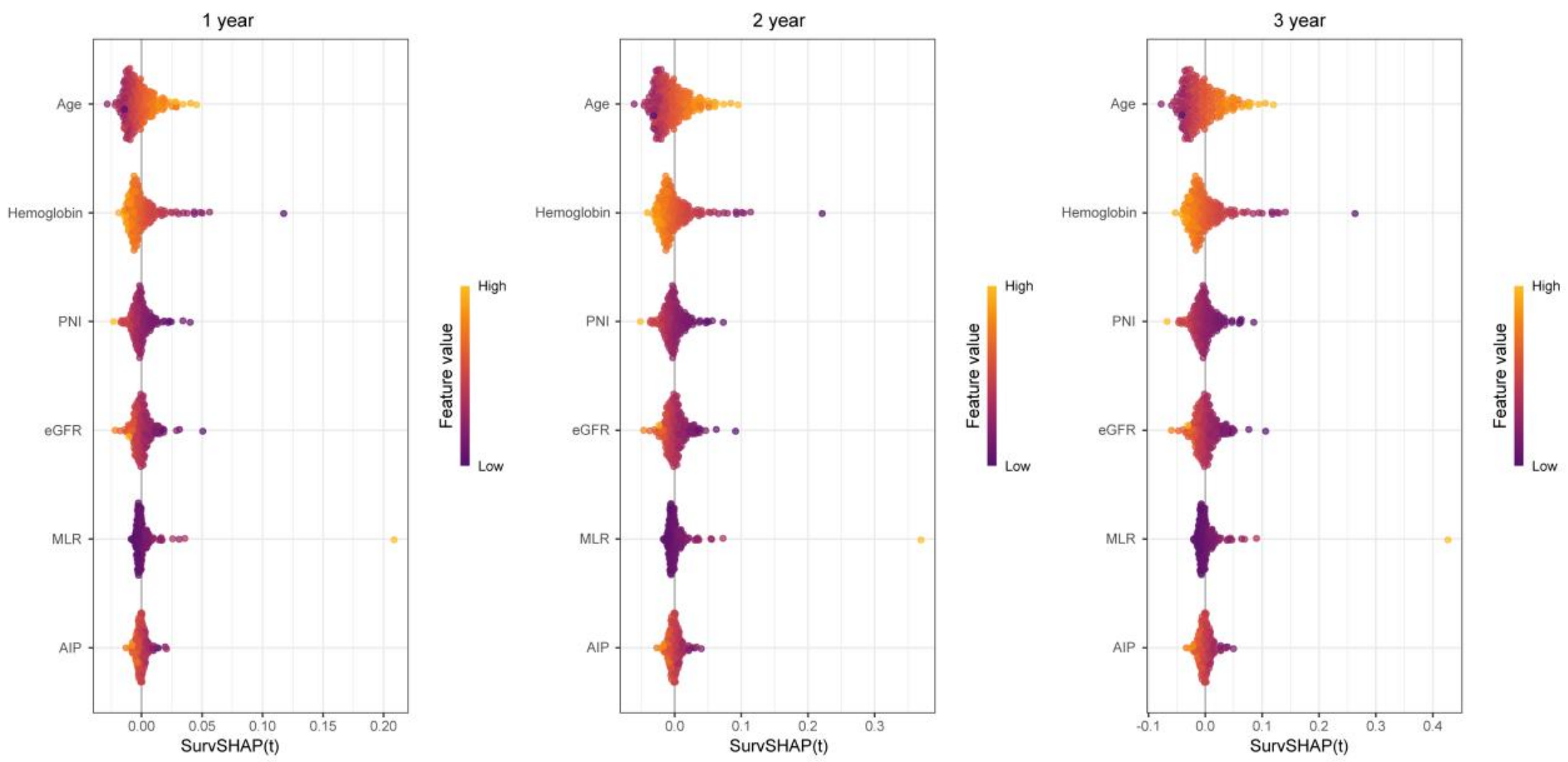

Supplementary Figure 8. Time-Dependent SurvSHAP(t) Importance of Predictors in the Final Elastic-Net Cox Model

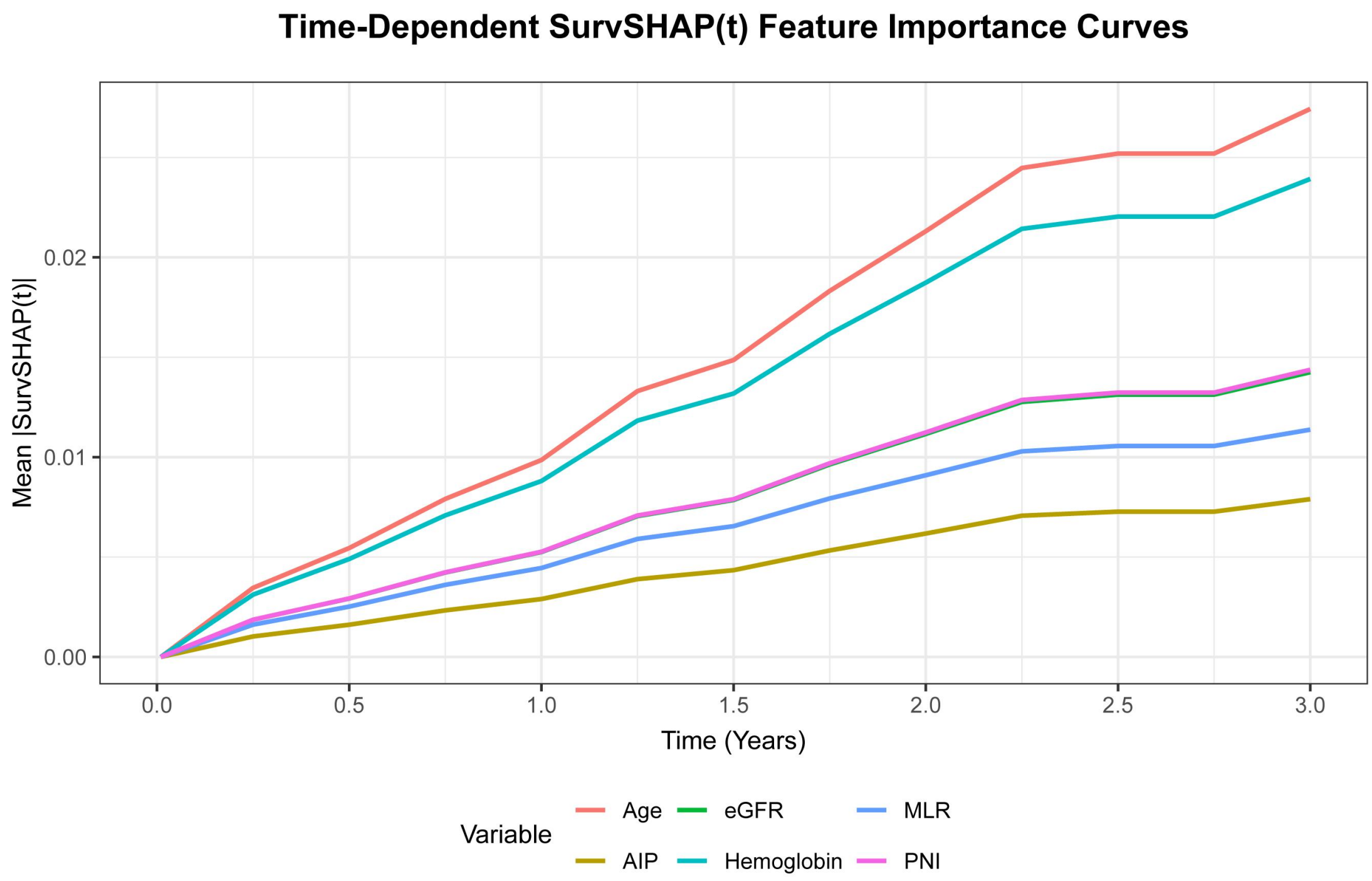

Supplementary Figure 9. Time-Dependent Permutation Feature Importance for the Final Elastic-Net Cox Model. A. Time-dependent permutation importance based on Brier loss for the elastic-net Cox model. B. Time-dependent permutation importance based on C/D AUC loss for the elastic-net Cox model

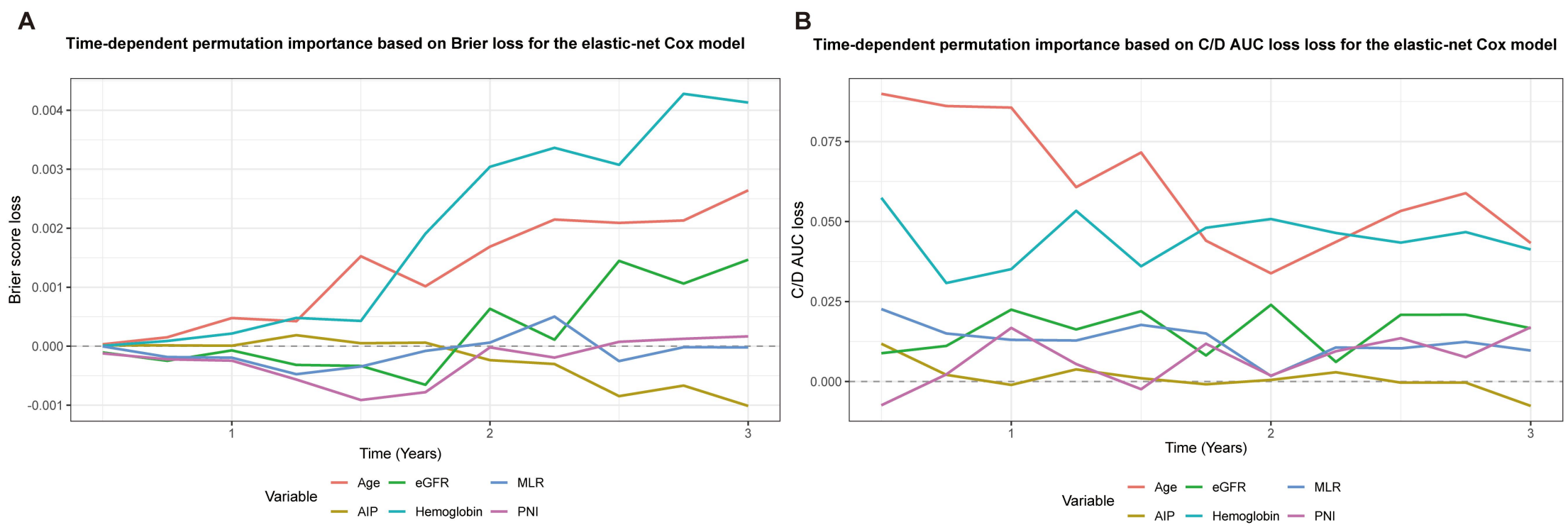

Supplementary Figure 10. Time-dependent survival partial dependence plots (PDPs) for the six predict

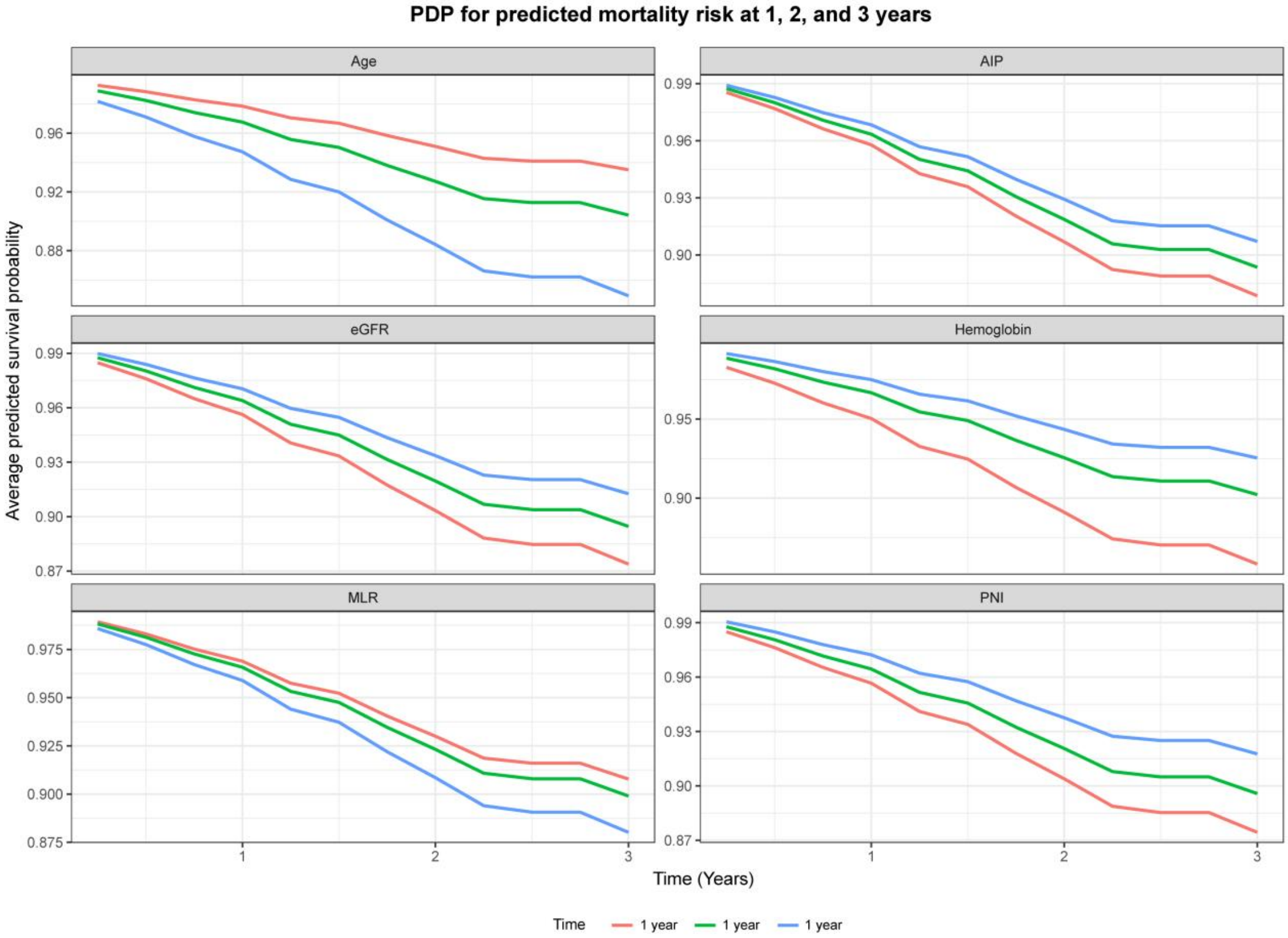

ors in the final elastic-net Cox model.

Supplementary Figure 11. Patient-level SurvSHAP(t) explanations over 0–3 years for a representative patient.

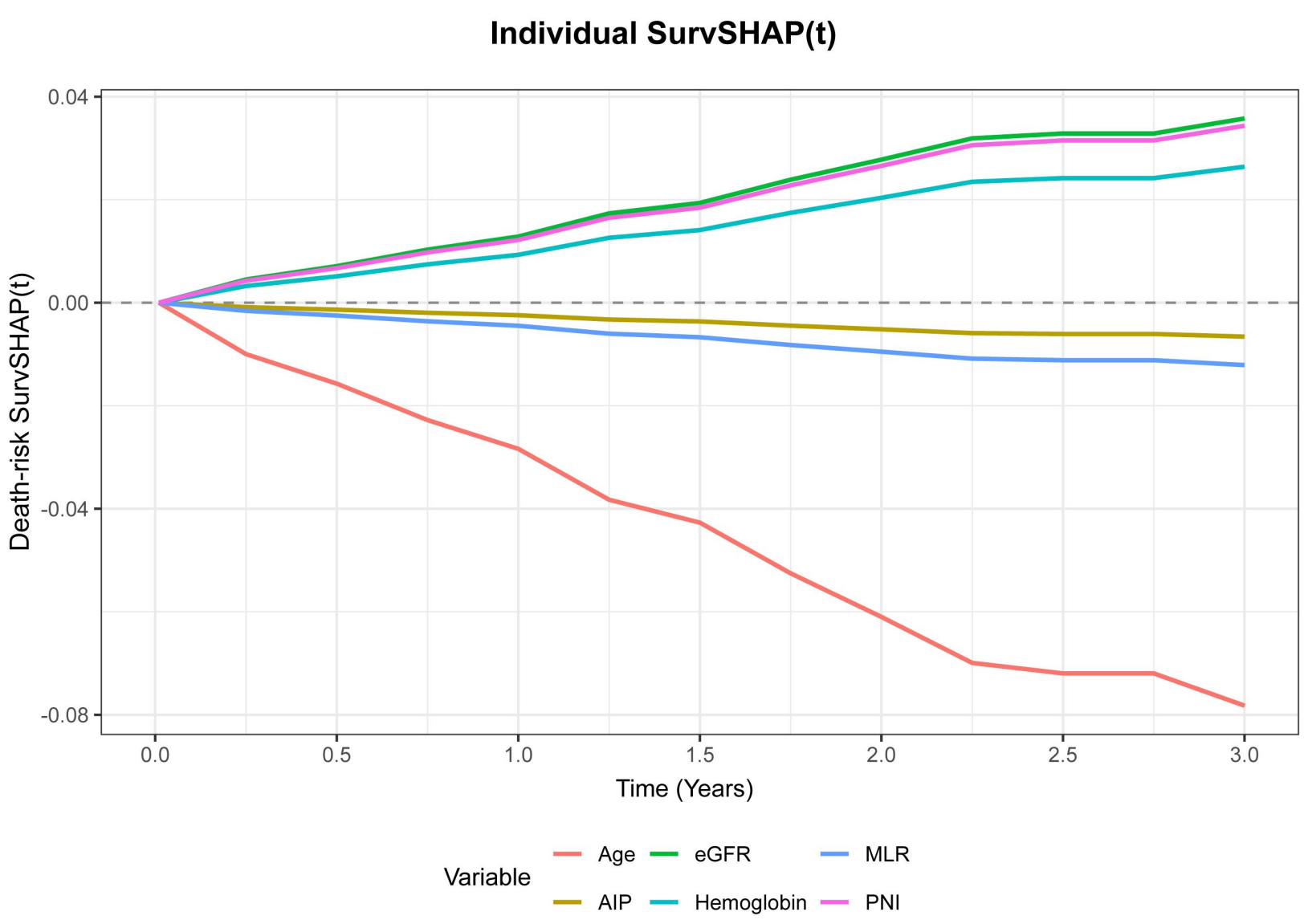

Supplementary Figure 12. Three-year calibration curve comparison between the final elastic-net Cox model and the GRACE risk score.

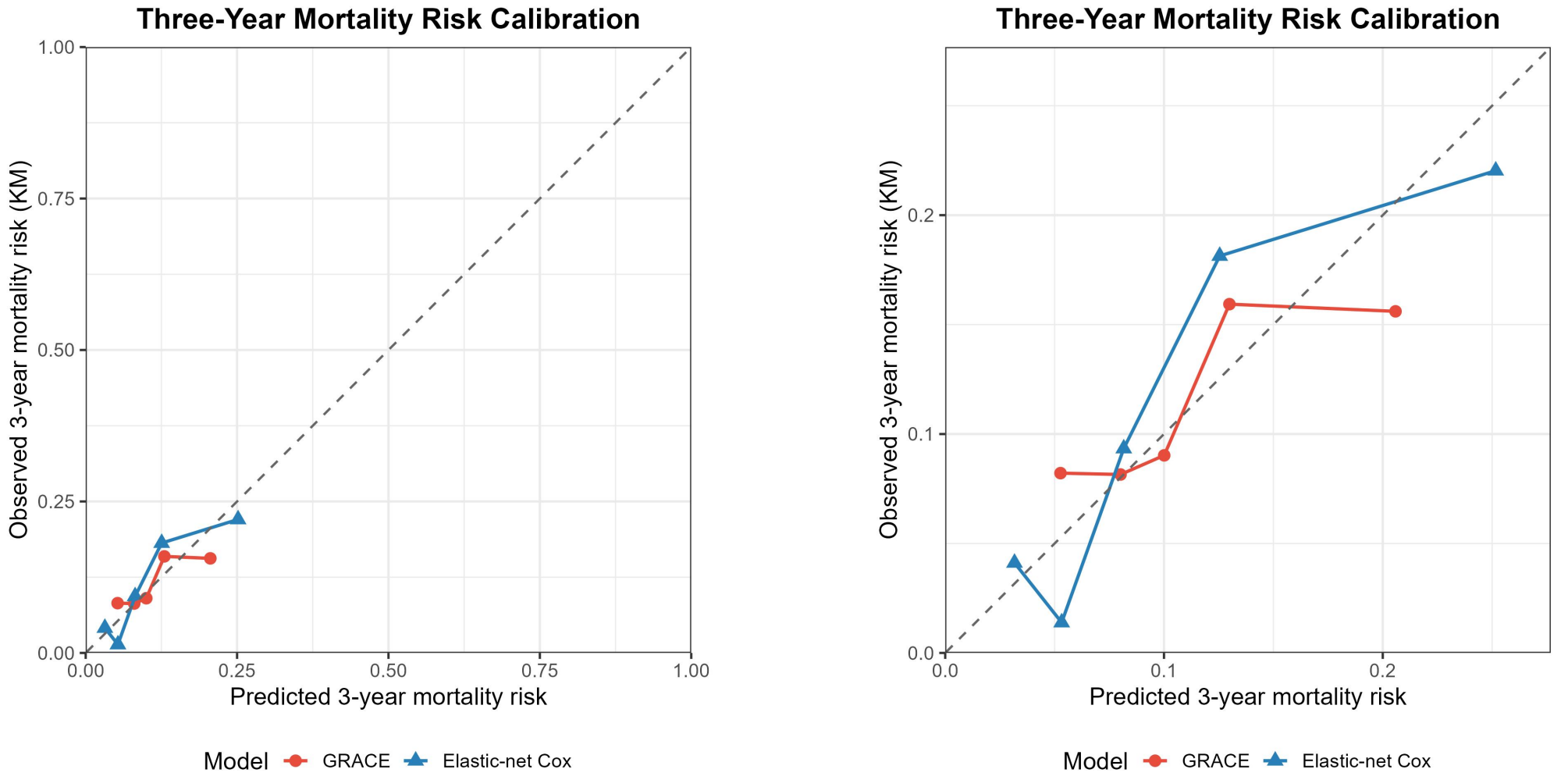

Supplementary Figure 13. Three-year decision curve analysis (DCA) comparing the final elastic-net Cox model with the GRACE risk score.

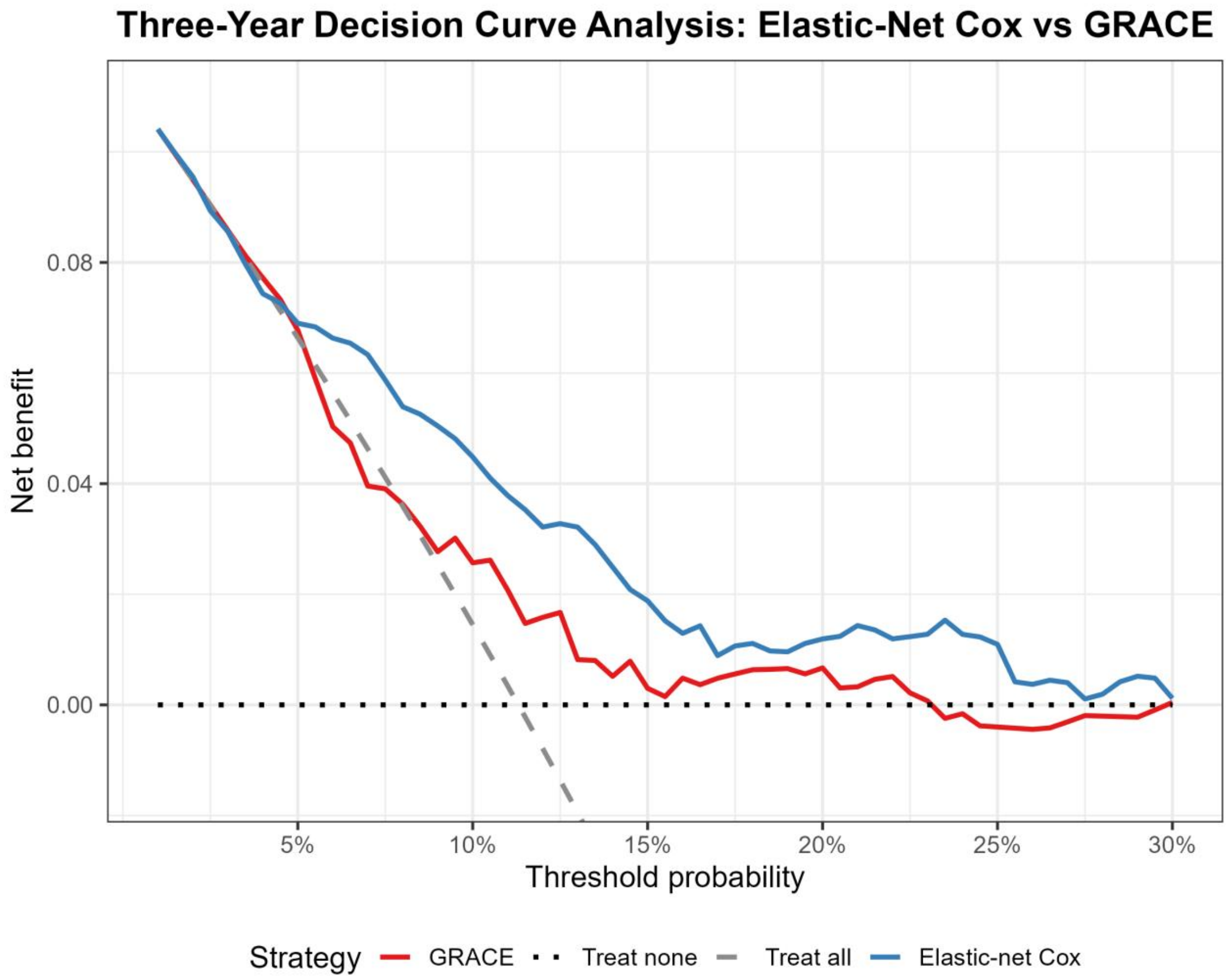

Supplementary Figure 14. Six-month ROC curve comparison between the final elastic-net Cox model and the GRACE risk score.

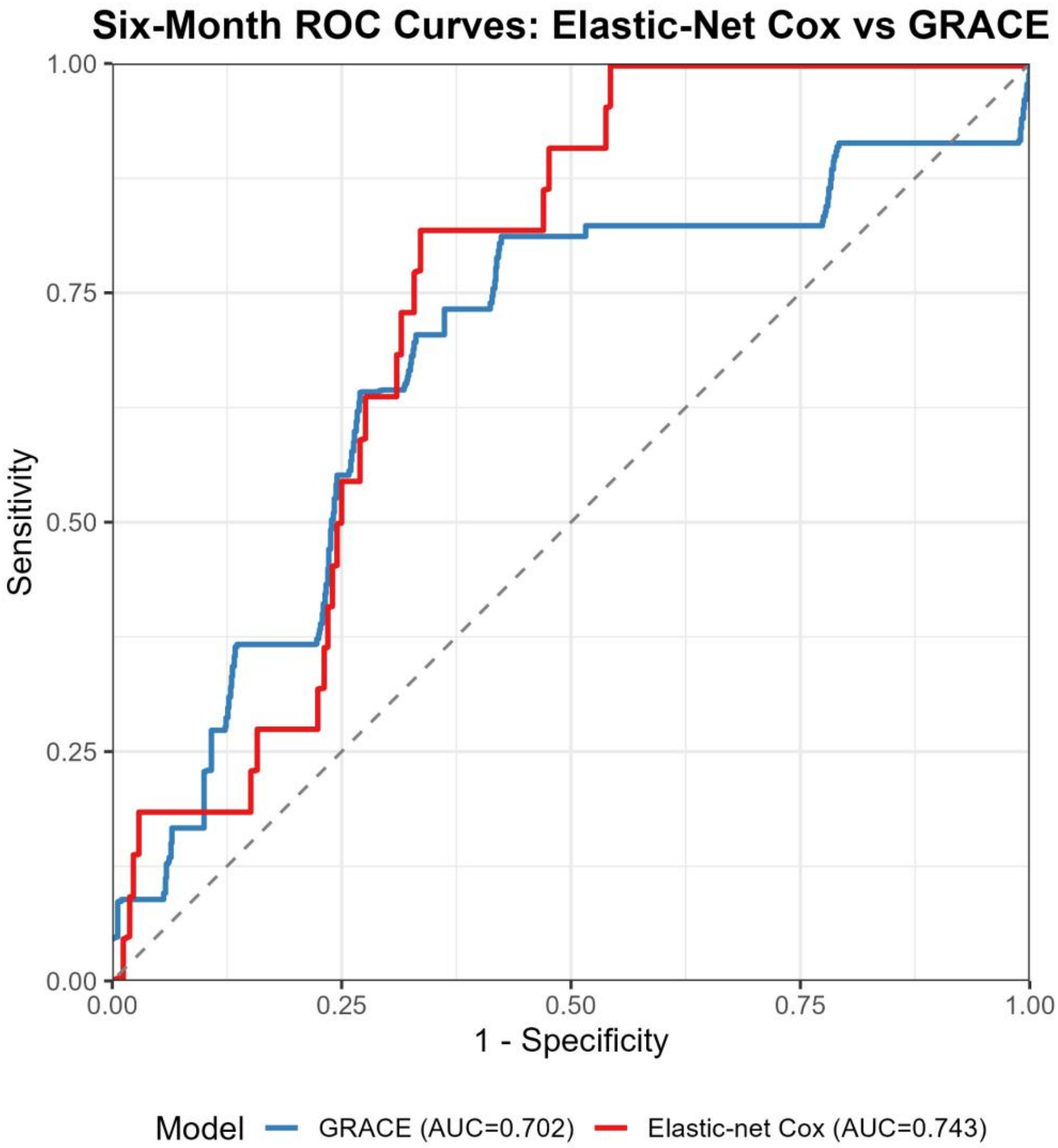

Supplement: Supplementary file 1 [file Datasheet1.pdf]
